# Investigation of Visual System Involvement in Spinocerebellar Ataxia Type 14

**DOI:** 10.1007/s12311-020-01130-w

**Published:** 2020-04-27

**Authors:** Thomas Ihl, Ella M. Kadas, Timm Oberwahrenbrock, Matthias Endres, Thomas Klockgether, Jan Schroeter, Alexander U. Brandt, Friedemann Paul, Martina Minnerop, Sarah Doss, Tanja Schmitz-Hübsch, Hanna G. Zimmermann

**Affiliations:** 1 Experimental and Clinical Research Center, Max Delbrück Center for Molecular Medicine and Charité – Universitätsmedizin Berlin, corporate member of Freie Universität Berlin, Humboldt-Universität zu Berlin and Berlin Institute of Health, Berlin, Germany; 2 NeuroCure Clinical Research Center, Charité – Universitätsmedizin Berlin, corporate member of Freie Universität Berlin, Humboldt-Universität zu Berlin and Berlin Institute of Health, Berlin, Germany; 3 Department of Neurology, Charité – Universitätsmedizin Berlin, corporate member of Freie Universität Berlin, Humboldt-Universität zu Berlin and Berlin Institute of Health, Berlin, Germany; 4 German Center for Neurodegenerative Diseases (DZNE), partner site, Berlin, Germany; 5 Department of Neurology, University Hospital of Bonn, Bonn, Germany; 6 German Center for Neurodegenerative Diseases (DZNE), Bonn, Germany; 7 University Tissue Bank, Cornea Bank Berlin, Institute of Transfusion Medicine, Charité – Universitätsmedizin Berlin, corporate member of Freie Universität Berlin, Humboldt-Universität zu Berlin and Berlin Institute of Health, Berlin, Germany; 8 Department of Neurology, University of California, Irvine, CA USA; 9 Institute of Neuroscience and Medicine (INM-1), Research Centre Juelich, Juelich, Germany; 10 Department of Neurology, Center for Movement Disorders and Neuromodulation, Medical Faculty, Heinrich-Heine University, Düsseldorf, Germany; 11 Department of Neurology and Institute of Clinical Neuroscience and Medical Psychology, Medical Faculty, Heinrich-Heine University, Düsseldorf, Germany; 12 Department of Neurological Sciences, Movement Disorders Section, University of Nebraska Medical Center, Omaha, NE USA

**Keywords:** Spinocerebellar ataxias, Protein kinase C gamma, Optical coherence tomography, Vision disorders

## Abstract

Spinocerebellar ataxia type 14 (SCA-PRKCG, formerly SCA14) is a rare, slowly progressive disorder caused by conventional mutations in protein kinase Cγ (PKCγ). The disease usually manifests with ataxia, but previous reports suggested PRKCG variants in retinal pathology. To systematically investigate for the first time visual function and retinal morphology in patients with SCA-PRKCG. Seventeen patients with PRKCG variants and 17 healthy controls were prospectively recruited, of which 12 genetically confirmed SCA-PRKCG patients and 14 matched controls were analyzed. We enquired a structured history for visual symptoms. Vision-related quality of life was obtained with the National Eye Institute Visual Function Questionnaire (NEI-VFQ) including the Neuro-Ophthalmic Supplement (NOS). Participants underwent testing of visual acuity, contrast sensitivity, visual fields, and retinal morphology with optical coherence tomography (OCT). Measurements of the SCA-PRKCG group were analyzed for their association with clinical parameters (ataxia rating and disease duration). SCA-PRKCG patients rate their vision-related quality of life in NEI-VFQ significantly worse than controls. Furthermore, binocular visual acuity and contrast sensitivity were worse in SCA-PRKCG patients compared with controls. Despite this, none of the OCT measurements differed between groups. NEI-VFQ and NOS composite scores were related to ataxia severity. Additionally, we describe one patient with a genetic variant of uncertain significance in the catalytic domain of PKCγ who, unlike all confirmed SCA-PRKCG, presented with a clinically silent epitheliopathy. SCA-PRKCG patients had reduced binocular vision and vision-related quality of life. Since no structural retinal damage was found, the pathomechanism of these findings remains unclear.

**Electronic supplementary material:**

The online version of this article (10.1007/s12311-020-01130-w) contains supplementary material, which is available to authorized users.

## Introduction

The spinocerebellar ataxias (SCA) denote the group of autosomal dominantly inherited ataxias clinically characterized by cerebellar syndrome and non-cerebellar involvement of varying degrees [1, 2]. The first genetically identified SCAs were caused by repeat-expansion mutations, and their phenotypes have been well described in large cohort studies (e.g., EUROSCA) [3–6]. SCA-PRKCG (formerly SCA14) was one of the first SCAs in which a conventional mutation was described as causative [7]. Its estimated incidence is 1 to 4% in ataxia populations with the more common SCA genotypes excluded [8]. SCA-PRKCG displays a considerable variability in age of onset. However, as life span is not shortened and the majority of people remain ambulatory until senectitude, it is considered a mild, slowly progressive disorder with few non-ataxia symptoms [9]. The protein kinase C gamma (PKCγ) is only expressed in neurons of the brain and the spinal cord and is particularly abundant in Purkinje cells [10]. Nonetheless, it also exhibits distinct dissemination in the visual system [11–15], especially in the human retina [14, 15]. In postmortem human retinas, PKCγ could only be stained in one rare type of ganglion cells and two different amacrine cell types [16]. PKCγ fulfills various functions in the visual system; for instance, it is an oxidative stress sensor in the lens and retina [12, 17], and it regulates rod photoreceptor differentiation [18] and plays an essential role in the signaling cascade of the vascular endothelial growth factor (VEGF)–dependent retinal neovascularization [19]. Additionally, PKCγ-knockout mice show an enlarged ganglion cell and inner nuclear layer, which was histologically caused by empty spaces between cells [17].

Reduced vision-related quality of life has been observed in the most common SCAs [20]. Visual impairment in SCAs may be an expression of oculomotor dysfunction which can occur as part of the cerebellar syndrome or sign of extra-cerebellar involvement [21, 22]. Furthermore, disease-specific involvement of the afferent visual system has been shown in some SCAs, most prominently in SCA-ATXN7 (formerly SCA7) where cone-rod-dystrophy is part of the clinical syndrome [23]. Also, more subtle changes like reduced visual acuity or retinal changes have been shown in some SCAs [24–26]. While PRKCG had been assumed to be the locus of autosomal dominant retinitis pigmentosa type 11 (Rp11) by Al-Maghtheh et al., further analysis could rule out a pathogenic role of PRKCG in Rp11 [27, 28]. We therefore aimed to comprehensively investigate visual impairment and affection of the afferent visual system in SCA-PRKCG. Data on visual function in SCA-PRKCG have to date only been reported in three affected members of one SCA-PRKCG family who had been evaluated using visually evoked potentials (VEP) and funduscopy. While funduscopy depicted several regions of retinal degeneration in one eye of a 21-year-old girl, these findings were not consistent with an inherited retinal degeneration and not seen in the two other family members [29].

The National Eye Institute Visual Function Questionnaire (NEI-VFQ) is a well-established questionnaire to assess clinically important aspects of vision-related quality of life [30] and has shown good correlation to well-recognized endpoints in ophthalmologic and neurologic diseases such as multiple sclerosis or neuromyelitis optica spectrum disorders [31, 32]. NEI-VFQ revealed a significant reduction in vision-related quality of life in patients suffering from SCA-ATXN1, SCA-ATXN3, or SCA-CACNA1A. Visual impairment was not only evident regarding NEI-VFQ composite score but also in most of the subscores [20].

Optical coherence tomography (OCT) has been used as a non-invasive and easily applicable tool to evaluate structural changes of the retina and optic nerve in a wide spectrum of neurodegenerative diseases including the most common trinucleotide expansion SCAs, particularly in SCA-ATXN1 [33], SCA-ATXN3 [34], SCA-ATXN7 [23], or a mixed SCA cohort (SCA-ATXN1,2,3 or SCA-CACNA1A) [26]. The method has a spatial resolution comparable with histological preparations [35], can detect subtle changes in retinal architecture, and is now considered an early biomarker in various neuroinflammatory and neurodegenerative diseases [36–39]. Furthermore, OCT may be a powerful tool in differentiating diseases with similar clinical characteristics or work as a surrogate marker for disease stage and severity [40–43]. The aim of this study was to investigate visual function as well as structural change in the afferent visual system in patients with SCA-PRKCG compared to age-matched controls.

## Methods

### Ethics Statement

The study was conducted in accordance with the Declaration of Helsinki in its currently applicable version and applicable German laws. It was approved by the ethics committee of Charité Universitätsmedizin Berlin and all participants gave informed written consent.

### Participants

Data were derived from a multimodal, cross-sectional, multicenter, observational study, in which patients and healthy controls (HCs) of comparable age and sex were recruited to better characterize the clinical profile of SCA-PRKCG. Details were described elsewhere [44, 45]. The study considered patients aged > 18 years with ataxia and a variant in the PRKCG gene. For inclusion into the analysis, variants had to be re-classified by protein modeling as at least likely pathogenic using a classification approach modified from Richards et al. [45, 46]. HCs of similar age were recruited among the patients’ environment or were other volunteers.

**Fig. 1:**
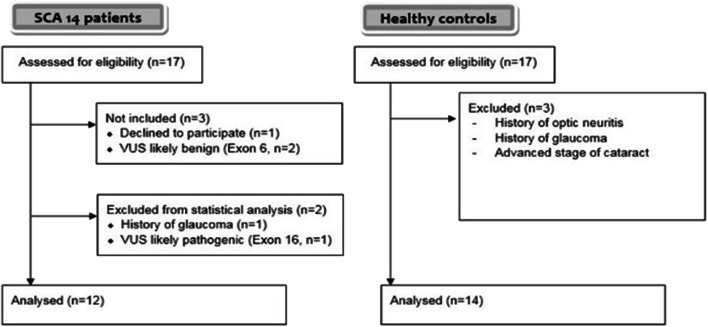
Inclusion and exclusion of patients. Participants were recruited from a multimodal, cross-sectional, multicenter, observational study. Participants with known current ophthalmologic diseases were excluded. Only patients with verified mutations in PRKCG were included for statistical analysis

For the visual substudy, we screened 17 patients with ataxia and PRKCG variant and 17 HC between November 2012 and April 2014. For analysis, we excluded participants with a known history of ophthalmologic diseases that could potentially influence the OCT and visual outcomes. Two ataxia patients were excluded because their variants were re-classified as benign. We report in the supplement the results of one patient who carried a variant of uncertain significance (VUS) in the kinase domain of SCA-PRKCG and one other confirmed SCA-PRKCG patient with glaucoma. Reasons for exclusion are summarized in Fig. 1.

### Clinical Examination

All tests were carried out by trained examiners within 1 day per participant. Structured history taking covered the need of refractive error correction and conditions potentially interfering with visual assessment (diabetes, arterial hypertension, cataract, glaucoma).

Clinical examination was performed by trained movement disorder specialists and included the 8-item scale for assessment and rating of ataxia (SARA) [47] to evaluate ataxia severity and the oculomotor items of the inventory of non-ataxia signs (INAS) [48], oscillopsia, and onset and severity of diplopia. Disease onset was defined as the onset of progressive gait disturbance.

### Vision-Related Quality of Life

Every participant completed the German version of the “National Eye Institute – Visual Function Questionnaire” (NEI-VFQ) in a self-administered manner [49]. This includes the 25 basic questions of NEI-VFQ as well as the 14 questions of the appendix and the 10-Item Neuro-Ophthalmic Supplement (NOS) developed by Raphael et al. [50]. Subscales include general health, general vision, well-being, ocular pain, near-, distance-, color-, and peripheral vision, social functioning, driving, role-limitation, and dependency whereas the NOS focuses on difficulties due to visual field defects, unusual eyelids, blurred vision/diplopia, and different vision of both eyes. Test results were recoded and subscores were averaged as described in detail in the NEI-VFQ manuals [51, 52] or according to the guidelines for NOS published by Wagenbreth et al. [53], respectively. The NOS composite score represents the unweighted average of all ten NOS items, while the total composite score is the result of the unweighted average of all ten NOS items and 24 items of NEI-VFQ, except the “general health” score. When participants gave an invalid answer to an item (no answer, two answers, not able to answer the question), the respective subscore was computed only for the participants who gave correct answers.

### Visual Function Testing

Visual acuity and contrast sensitivity were assessed on the OPTEC 6500 P system (Stereo Optical Co, Inc., IL, USA) under habitual correction using photopic conditions (target luminance value = 85 cd/m^2^) without glare. Early Treatment Diabetic Retinopathy Study (ETDRS) charts were used for high-contrast visual acuity (HCVA) testing and Functional Acuity Contrast Test (FACT) for contrast sensitivity testing. HCVA was tested both monocular and binocular, and contrast sensitivity only binocular as this is suggested to represent the every-day vision [54, 55]. HCVA results were converted into a logarithm of the minimal angle of resolution scores (logMAR). Contrast sensitivity results were expressed as area under the curve as described earlier [56].

### Visual Field Analysis

Monocular visual fields were assessed by automated Humphrey Field Analyzer II 720 (HFA, Zeiss Meditec AG) with integrated automated Glaucoma Hemifield Test (GHT) using the 30-2 protocol.

Because of poor reliability indices in the ataxia cohort, we do not report visual field parameters in the results.

### Optical Coherence Tomography

All participants underwent retinal examination with a Spectral-Domain-OCT (Spectralis, Heidelberg Engineering, Heidelberg, Germany) with an integrated scanning laser ophthalmoscope (SLO) and activated eye tracker. Scans were carried out without pupil dilatation by four operators always using the same device with normal room illumination.

**Fig. 2:**
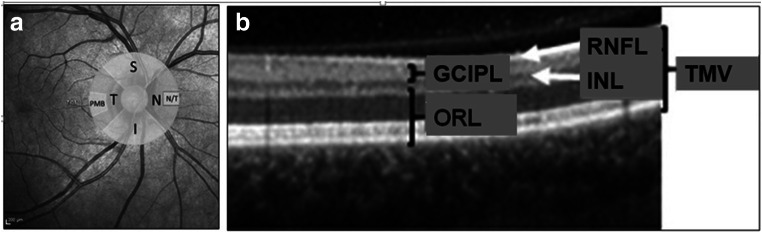
Illustration of OCT parameters. **a** Peripapillary ring scan. Peripapillary ring scan measures retinal nerve fiber layer (RNFL) thickness within the whole circle, in the quadrants S(uperior), N(asal), I(nferior), and T(emporal) and in the papillomacular bundle (PMB). The ratio of nasal to temporal RNFL thickness (N/T ratio) is calculated automatically. **b** Layer segmentation analysis. Layer segmentation analysis was performed within a 6-mm-diameter circle around the fovea. We measured thickness of the following layers: RNFL (mRNFL) (macular), the combined ganglion cell and inner plexiform layer (GCIPL), and the inner nuclear layer (INL) and outer retinal layers (ORL) including all layers from the outer plexiform layer to the Bruch membrane. The sum of all these layers adds up to the total macular volume (TMV).

For peripapillary retinal nerve fiber layer (pRNFL) thickness measurements, a 3.4-mm diameter ring scan (12°, 1536 A-scans, 16 ≤ ART≤ 100) centered on the optic nerve head was used. We analyzed the global thickness of the ring, and the thickness of the four quadrants, nasal, temporal, superior, inferior, and the papillomacular bundle as well as the nasal to temporal pRNFL thickness (N/T) ratio. Ring scan parameters are illustrated in Fig. 2a.

Furthermore, a macular volume scan was assessed (25°×30°, 61 vertical B-scans, 768 A-scans per B-scan, ART = 12). Macular scans underwent automatic intra-retinal segmentation with software provided by the manufacturer (Eye Explorer 1.8.6.0 with viewing module 5.8.3.0). Besides the total macular volume, the combined ganglion cell, and the inner plexiform layer, the inner nuclear layer as well as outer retinal layers including all layers from the outer plexiform layer to the Bruch membrane were extracted within a 6-mm-diameter circle around the fovea. Figure 2b depicts all analyzed layers.

All scans were carefully checked for sufficient scan quality and accurate segmentation as described elsewhere [57] by one experienced grader, who was blinded for the group. All images met the OSCAR IB criteria for image quality [57]. Segmentation was manually corrected if necessary.

OCT images and their corresponding scanning laser ophthalmoscope fundus images were screened for abnormalities or pathologies by experienced physicians and optometrists. Patients with abnormal image features were additionally evaluated by an ophthalmologist. OCT results were reported according to the APOSTEL recommendations [58].

### Statistical Analysis

Statistical analysis was carried out using SPSS 22 (IBM, Armonk, NY) software. Demographic differences between patients and HC were analyzed using an independent sample Mann-Whitney *U* test (MWU) for age and a 2-sided chi^2^ test for sex. NEI-VFQ and NOS analysis included descriptive data analysis and MWU for between-group comparisons. The beeswarm plots were created using R software version 3.6.1, RStudio Version 1.2.1335, and ggplot2/ggpubr as data visualization packages. We used generalized estimation equation models (GEEs) accounting for within-subject inter-eye effects for group comparisons of monocular visual acuity and OCT. The working correlation matrix was defined as exchangeable. In the second step of the analysis, we investigated associations of disease severity (SARA score) with quality of life scores and results of binocular visual acuity test using Spearman’s rho test. We further analyzed a possible association of ataxia severity to each OCT measure using GEE which takes into account the inclusion of individual data from both eyes. A *p* value < 0.05 was established as significant. We made no adjustments for multiple comparisons due to the exploratory nature of the study.

## Results

After consideration of exclusion criteria, twelve SCA-PRKCG patients (9 members of 4 families and 3 singular cases) and 14 HC were available for analysis (Fig. 1). All patients exhibited mutations in exon 1–4, i.e., within the regulatory domain of PRKCG. Demographic, clinical, and functional visual outcomes are presented in Table 1. Groups did not differ in age or sex. A more detailed overview of the individual results regarding patient history, visual acuity test, fundus imaging, and clinical parameters (disease duration/severity, variant) is provided in the supplement (supplement Table 1; supplement Table 2 lists all individual OCT results).

**Table 1:** Cohort overview and functional visual outcomes

	SCA-PRKCG	HC	*p*
Total	12	14	
Sex			
Male	6	8	0.716 (Chi^2^)
Female	6	6	
Age/years			
Mean ± SD	51.75 ± 13.6	50.7 ± 11.1	0.820 (MWU)
Range	29–70	30–65	
Disease duration			
Years, mean ± SD	20.7 ± 13.7		
Range	0–44		
SARA score			
Mean ± SD	11.4 ± 3.6		
Range	6.5–20.5		
Visual acuity			
Logmar score monocular ± SD	0.04 ± 0.14	− 0.02 ± 0.10	0.137 (GEE)
Logmar score binocular ± SD	− 0.03 ± 0.12	− 0.13 ± 0.82	*0.036* (MWU)
FACT AULCSF binocular ± SD	1.92 ± 0.15	2.11 ± 0.06	*p* < 0.001 (MWU)

Values for both groups are mean ± standard deviation. Significant *p* values are printed in italics

*AULCSF* area under the log contrast sensitivity function, *HC* healthy control, *FACT* Functional Acuity Contrast Test, *GEE* generalized estimation equation models, *logMAR* logarithm of the minimal angle of resolution, *MWU* Man-Whitney *U* test, *SARA* scale for the assessment and rating of ataxia, *SCA-PRKCG* spinocerebellar ataxia type 14, *SD* standard deviation

In the self-reported visual history, one patient reported an eye infection of unknown etiology earlier in his life and another patient reported ocular pain of unknown origin. In the clinical examination, all patients displayed saccadic eye movements and the majority had dysmetric saccades while some sort of gaze-evoked nystagmus was less frequently seen. Deficits in the vestibulo-ocular reflex (VOR) were only seen in two patients while nearly all patients had a disturbed VOR suppression on fixation. Seven out of 12 patients suffered from diplopia rated as moderate in two and as severe/constant in one patient. Some difficulty in horizontal or vertical gaze was seen in two SCA-PRKCG patients, though one of them was excluded from analysis due to glaucoma.

**Fig. 3:**
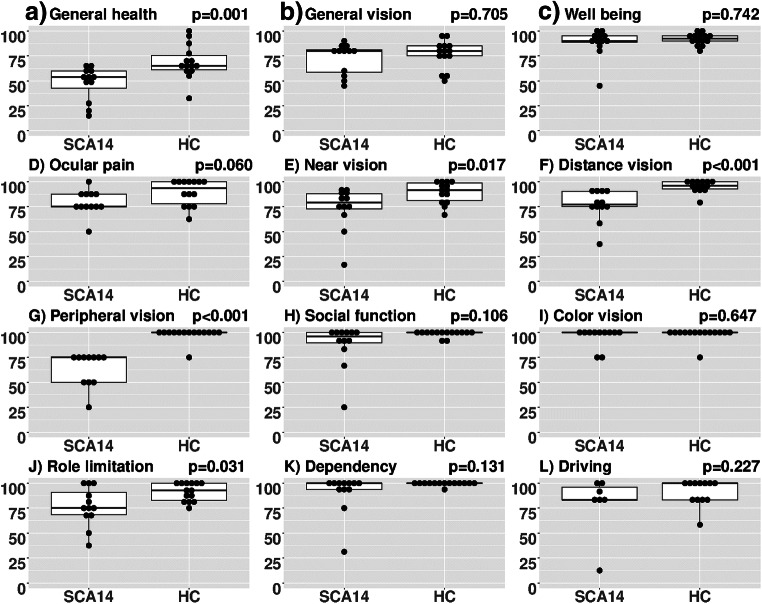
**a**–**l** Results of NEI-VFQ and Neuro-Ophthalmologic Supplement. Comparison of vision-related quality of life results in all NEI-VFQ subscales between SCA-PRKCG and HC. The *y*-axis represents the result of the particular NEI-VFQ subscore, each scored in a range of 0–100. NEI-VFQ, National Eye Institute Visual Function Questionnaire; SCA-PRKCG, spinocerebellar ataxia type 14; HC, healthy control

As a group, patients had a significantly worse vision-related quality of life in NEI-VFQ compared to HC. Their results were particularly poor for near, distance, and peripheral vision. Figure 3 visualizes the results of all NEI-VFQ subscores. In the NOS, patients reported more difficulties when eyes are tired than HC (*p* = 0.004), while no other NOS subscore reached significance. NOS subscores together with the composite scores of NEI-VFQ, NOS, and a total composite score of both are summarized in Table 2.

**Table 2:** Neuro-Ophthalmic Supplement (NOS) and composite scores

NOS subscale	SCA-PRKCG (*n* = 12)	HC (*n* = 14)	*p*
Difficulty when eyes tired	56.3 ± 21.7	80.3 ± 14.5	*0.004*
Difficulty in bright sunlight	72.9 ± 34.5	87.5 ± 13.0	0.494
Difficulty parking a car	63.9 ± 48.6/*n* = 9	83.9 ± 36.2	0.369
Difficulty using a computer	83.3 ± 16.3	94.6 ± 10.6	0.095
Two eyes see different	58.3 ± 41.7	78.6 ± 27.5	0.274
Eyelid appearance unusual	87.5 ± 22.6	92.9 ± 18.2	0.667
Vision blurry, not clear, “fuzzy”	81.3 ± 15.5	91.1 ± 12.4	0.145
Trouble focusing on moving objects	79.2 ± 27.9	98.2 ± 6.7	0.060
Binocular double vision	70.8 ± 36.7	96.4 ± 9.1	0.085
Ptosis	95.8 ± 9.7	92.9 ± 26.7	0.742
10-item NOS composite score	75.1 ± 18.9	89.6 ± 8.9	*0.013*
NEI-VFQ composite score	79.4 ± 15.1	92.6 ± 4.2	*< 0.001*
Total composite score	77.3 ± 16.6	91.0 ± 5.1	*0.001*

Values for both groups are means ± standard deviation. Significant *p* values (resulting from the Mann-Whitney *U* test) are printed in italics.

*HC* healthy control, *NOS* Neuro-Ophthalmic supplement, *NEI-VFQ* composite score: National Eye Institute – Visual Function Questionnaire composite score (without NOS), *Total composite score* composite of NEI-VFQ and NOS

Visual acuity testing revealed poorer results for SCA-PRKCG patients compared with that for HC in binocular visual acuity and binocular contrast sensitivity, while monocular test results showed no significant difference between groups.

The results of OCT parameters are summarized in Table 3. We compared pRNFL thickness of all quadrants and the papillomacular bundle in the peripapillary ring scan and layer thickness parameters of the ganglion cell and inner plexiform layer, inner nuclear layer, outer retinal layers, and total macular volume from the macular scans. Neither in peripapillary nor in macular analysis was there a significant difference between both groups.

**Table 3:** Retinal thickness measurements from OCT

Parameter	HC (*n* = 14), mean ± SD	Min–max	SCA-PRKCG (*n* = 12), mean ± SD	Min–max	*p* GEE
Peripapillary ring scan					
Average pRNFL (μm)	100.0 ± 6.3	89–114	101.8 ± 8.1	86–114	0.507
Superior pRNFL (μm)	117.1 ± 11.9	97–146	121.25 ± 15.4	92–151	0.369
Temporal pRNFL (μm)	72.0 ± 10.5	54–98	69.1 ± 13.3	52–112	0.455
Inferior pRNFL (μm)	130.1 ± 9.8	113–150	138.25 ± 16.0	115–178	0.082
Nasal pRNFL (μm)	80.8 ± 11.3	61–103	78.8 ± 12.0	54–104	0.606
RNFL-PMB (μm)	55.5 ± 7.0	44–69	53.3 ± 9.3	41–82	0.403
N/T ratio	1.15 ± 0.24	0.62–1.72	1.19 ± 0.33	0.62–1.77	0.635
Macular volume scan					
TMV (mm^3^)	8.75 ± 0.18	8.40–9.11	8.81 ± 0.31	8.00–9.38	0.594
mRNFL (μm)	36.7 ± 3.1	30.1–41.7	34.8 ± 2.8	30.1–39.3	0.085
GCIPL (μm)	70.0 ± 3.6	63.0–74.6	72.3 ± 6.2	61.9–84.5	0.263
INL (μm)	33.3 ± 2.3	29.7–38.6	34.3 ± 2.8	30.1–42.4	0.313
ORL (μm)	169.6 ± 6.9	156.3–183.2	170.1 ± 5.8	158.1–177.9	0.834

Values for both groups are mean ± standard deviation

*GCIPL* ganglion cell and inner plexiform layer, *INL* inner nuclear layer, *mRNFL* macular retinal nerve fiber layer, *N/T ratio* nasal to temporal ratio, *OCT* optical coherence tomography, *ORL* outer retinal layers from outer plexiform layer to Bruch’s membrane, *PMB* papillomacular bundle, *pRNFL* peripapillary retinal nerve fiber layer, *SD* standard deviation, *TMV* total macular volume

**Fig. 4:**
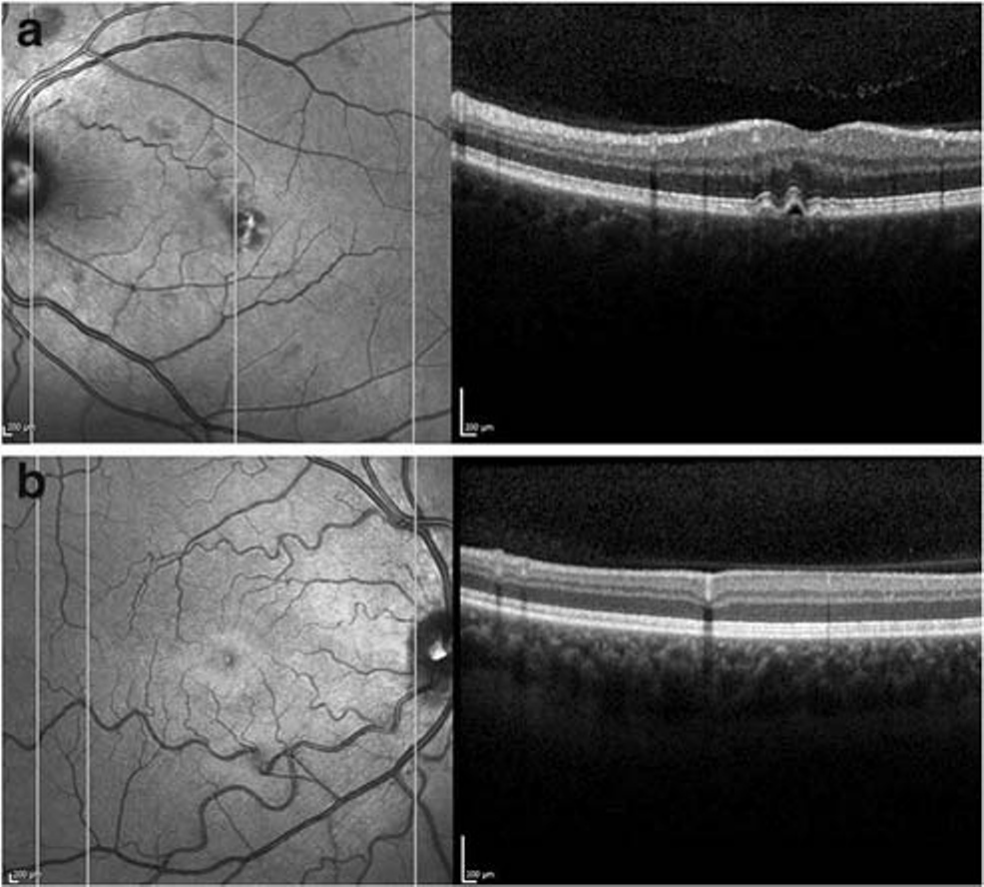
Retinal findings in two patients. **a** Scanning laser ophthalmoscope image (SLO) (left) and corresponding OCT cross-section (right) of the left eye of patient 16. Both eyes of this patient displayed an epitheliopathy of unknown origin. Note that this patient was excluded from statistical analysis because of unverified SCA-PRKCG diagnosis. **b** SLO image (left) and corresponding OCT cross-section (right) of patient 3, who showed marked tortuosity in both eyes. As this is the only patient of our cohort exhibiting unexplained tortuosity, we considered this not disease-associated

Among the 12 SCA-PRKCG patients, 3 had remarkable abnormalities in SLO fundus images (an overview is given in Supplement Table 1). Among those was one young patient who showed an unspecific but remarkable tortuosity of the retinal arteries in both eyes (Fig. 4a). The other findings were drusen and calcifying exudates which are both very common in elderly patients.

Remarkably, the single patient with a variant re-classified as VUS displayed a clinically silent epitheliopathy of unknown origin in both eyes (Fig. 4b) that was not seen in any of the confirmed SCA-PRKCG patients.

**Fig. 5:**
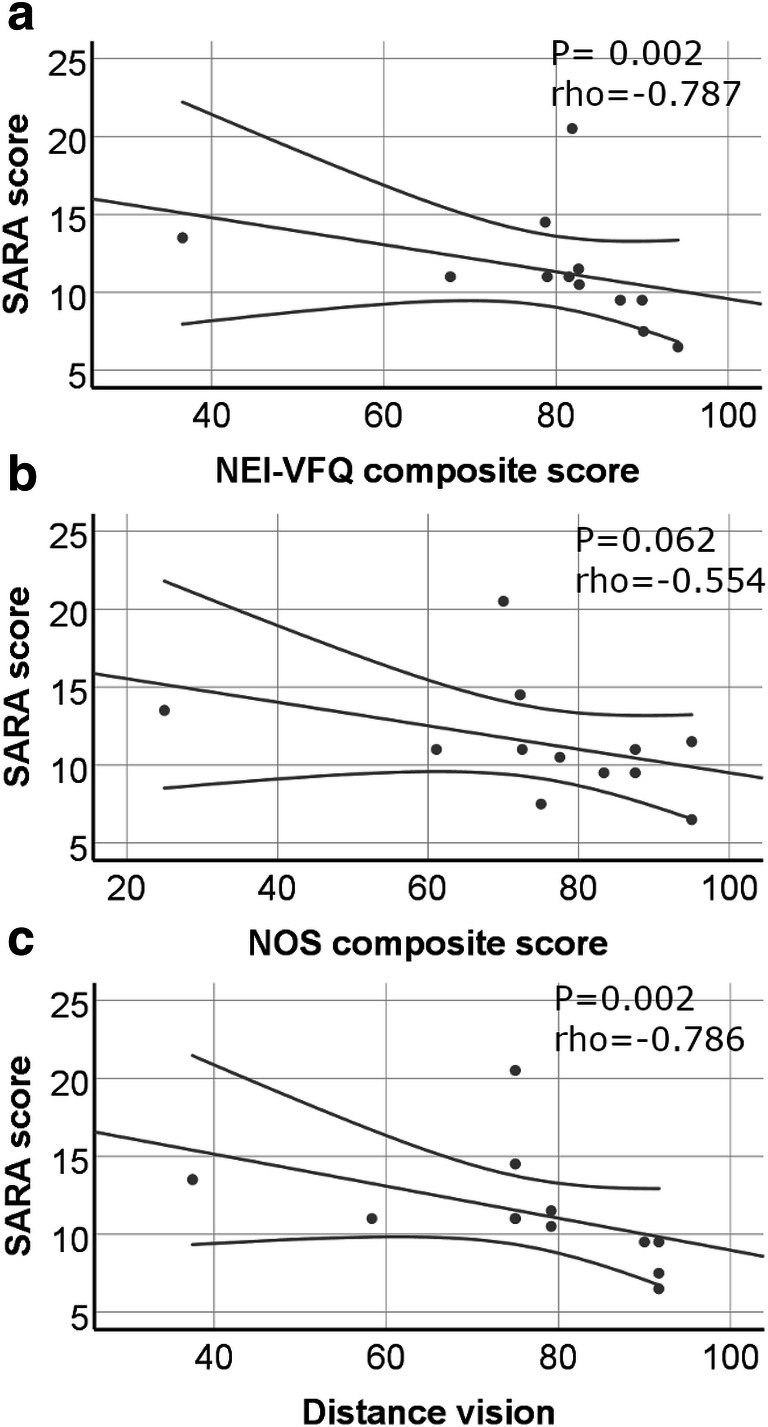
**a**–**c** Vision-related quality of life and disease severity. Disease severity (measured by SARA score) correlated inversely to both questionnaires for vision-related quality of life. Distance vision was especially worse in SCA-PRKCG patients and showed fair correlation to disease severity, too

**Fig. 6:**
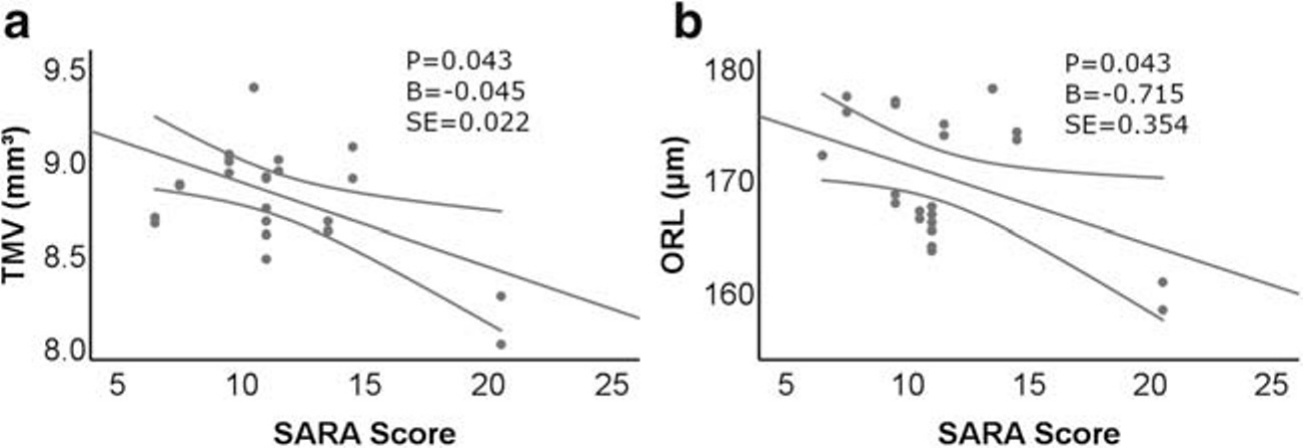
SARA scores and retinal layer thickness. Scatter plots for OCT measures versus SARA (ataxia severity) for those parameters that showed significant results in GEE linear modeling. Only total macular volume (TMV) and outer retinal layers (ORL) thickness were related to clinical disease severity SARA score), but significance depended on the single more severely ataxic subject. Statistics come from generalized estimating equations. B, non-standardized correlation coefficient; SE, standard error

Patients with higher ataxia scores had also a lower vision-related quality of life (rho = − 0.787, *p* = 0.002) (Fig. 5). Especially the subscores general vision (rho = − 0.641, *p* = 0.025), near vision (rho = − 0.715, *p* = 0.009), and distance vision (rho = − 0,786, *p* = 0.002) were worse in more severely affected patients. There was no association of disease severity neither to NOS scores nor to visual acuity (monocular/binocular HCVA and LCVA). Furthermore, these parameters of visual acuity did not show any significant correlation to NEI-VFQ scores. Exploratory analysis revealed only weak effects of ataxia severity scores with higher scores (more severe ataxia) related to thinner total macular volume (*p* = 0.043, *B* = − 0.045) and outer retinal layers (*p* = 0.043, *B* = − 0.715), whereas no effects were seen on pRNFL. However, the inspection of respective plots (Fig. 6) suggested that this was due to the lower values in the one (elderly) subject with high SARA score.

We found no associations between one distinct oculomotor deficit and low vision-related quality of life, high rate of fixation loss in visual field testing, or lower binocular visual acuity. Specifically, the patients with moderate to severe diplopia nevertheless had a low rate of fixation loss and good visual acuity results.

## Discussion

To the best of our knowledge, this is the first study to systematically evaluate visual system changes in patients with SCA-PRKCG. We found that (1) vision-related quality of life was lower in SCA-PRKCG patients compared with that in HC, particularly for the items near-, distance-, and peripheral vision. Furthermore, the impairment in vision-related quality of life was correlated with ataxia severity, measured by SARA score, which means that patients with higher SARA scores had a worse vision-related quality of life. (2) Visual acuity was worse in the SCA-PRKCG group regarding binocular high- and low-contrast visual acuity testing and none of the oculomotor deficits alone accounts for the results above. (3) We did not find significant changes in retinal layer thickness in SCA-PRKCG compared with HC. Exploratory analysis revealed only weak associations of higher SARA scores and lower total macular volume and outer retinal layers mainly based on one elderly individual with high SARA score. Thus, in contrast to other SCA genotypes, our results do not support retinal thinning or retinal dysfunction (as indicated by monocular testing of visual acuity) as a prevalent or disease-specific finding in SCA-PRKCG.

In order to explain the reduced (binocular) visual function in SCA-PRKCG patients, we checked the individual visual acuity and contrast sensitivity results. According to the International Council of Ophthalmology recommendations, only 3/12 patients had a “mild visual loss” in binocular visual acuity, defined as visual acuity value ≤ 0.8 decimal (logMAR score ≥ 0.1) [59] while none of the patients showed moderate visual acuity deficits. Because of the small sample size, we cannot rule out that group differences in visual acuity tests are based on a selection bias towards a HC group with above-average visual function. Non-significant age differences to the disadvantage of the SCA-PRKCG group may contribute to worse visual acuity results in the SCA group. Furthermore, testing was performed with habitual corrections. Thus, the slight differences in binocular visual acuity alone would not present a valid sign of visual impairment. However, there is a substantial and highly significant difference in binocular contrast sensitivity between groups (AULCSF, 1.92 ± 0.15 in SCA-PRKCG vs. 2.11 ± 0.06 in HC, *p* < 0.001). Individual contrast sensitivity results of PRKCG-patients are in the range of earlier studies in multiple sclerosis cohorts (with about half with a history of optic neuritis) [56]. Furthermore, results almost perfectly distinguish PRKCG patients from HC so that a mere effect of selection bias is unlikely.

From a clinical point of view, it seems most probable that reduced visual acuity on binocular testing is associated with lower vision-related quality of life (VRQoL). However, such correlations could not be confirmed in our small sample.

Hence, we will discuss three lines of argument as possible explanations for reduced (binocular) visual acuity and contrast sensitivity in SCA-PRKCG along with unimpaired monocular visual acuity: (1) technical issues, (2) affection of the visual pathway, and (3) an influence of the cerebellar disorder. Later on, we discuss factors influencing VRQoL independently.

Technical issues might have influenced visual acuity results. In our study, visual acuity testing was performed habitually and not under best correction. Therefore, a stronger influence of an uncorrected refractive error in the patient group is possible.

Despite taking patients’ history of ophthalmologic diseases, we cannot completely rule out that other ocular diseases such as glaucoma might have caused worse visual outcomes in the PRKCG cohort. However, we did exclude patients with a known history of glaucoma (one in both groups) and there was no RNFL thinning in the SCA-PRKCG suggestive of glaucoma. Furthermore, cognitive impairment and mood disorders are potential confounders for worse visual acuity tests both of which were prevalent in the SCA-PRKCG cohort. Visual acuity tests particularly depend on the information processing speed [60]. In fact, all patients with markedly low-contrast sensitivity scores had mood disorders or cognitive impairment to some degree (personal communication on the contents of [45]). We assume that the high rates of fixation loss and the concurrent low reliability indices in the visual field tests are indicators of reduced processing speed in SCA-PRKCG patients and that these problems may partially explain reduced contrast sensitivity and vision-related quality of life. Regarding statistical analysis, we do not believe that different modes of statistical evaluation, namely the MWU test as a non-parametric rank-sum test for binocular visual acuity on the one hand and the semiparametric GEE for the monocular visual acuity on the other hand, were responsible for the significant binocular but not monocular between-group differences in visual acuity. GEEs were performed to account for more than one inner-subject variable and increase the power of the statistic evaluation. Hence, GEEs are considered even more sensitive in detecting between-group differences than rank-sum tests.

With respect to visual pathway affection, we acquired OCT images. This did not reveal structural retinal changes in SCA-PRKCG. In addition, there was no significant difference in layer segmentation analysis in comparison to the HC group. More specifically, values in pRNFL were numerically even slightly higher in the SCA-PRKCG group. A non-significant difference in this range cannot be considered thickening and should not be overinterpreted given the small number of patients. However, it should be mentioned that the thickening of the pRNFL may indicate papilledema in an inflammatory affection of the optic nerve. Furthermore, PKCγ-knockout mice show an increased thickness of the inner nuclear layer and ganglion cell layer due to large vacuoles between cells, while the total number of nuclei was reduced in these layers [17], and vacuoles may not be visible with OCT. Moreover, retinas of these mice have an increased sensitivity for oxidative stress, which causes decreased outer nuclear layer thickness [17]. However, PKCγ-knockout mice are not considered a suitable model for SCA-PRKCG as a toxic gain of function (and not loss of function) is supposed to cause SCA-PRKCG. Unfortunately, the two histopathological reports of SCA-PRKCG to date did not report on retinal findings [61, 62]. Hence, although retinal atrophy due to SCA-PRKCG is highly unlikely according to our findings, there remains uncertainty about a possible increase of retinal layer thickness in SCA-PRKCG and its histopathological and functional correlates. Taken together, our OCT data rather rule out retinal thinning as a feature of SCA-PRKCG and differ from results of earlier studies in other SCA types: Stricker et al. detected pRNFL thinning of the temporal quadrant and visual acuity reduction in SCA-ATXN1 [33]. Pula et al. found reduced pRNFL thickness in SCA-ATXN2 and SCA-ATXN3 but not in SCA-ATXN1 and a reduced TMV in SCA-ATXN1, SCA-ATXN3, and SCA-CACNA1A [26]. Alvarez et al. described a reduced pRNFL thickness without a specific pattern of thinning in sector analysis in SCA-ATXN3. None of the studies has analyzed intra-retinal layer thickness of the macula. It is important to note that Pula et al. and Alvarez et al. reported a correlation between disease severity and pRNFL thinning for SCA-ATXN2 and SCA-ATXN3 or SCA-ATXN3 alone [26, 34], but data were limited to small sample sizes. Both studies did not report visual acuity for their cohort. In conclusion, studies evaluating vision-related quality of life indicate a reduction in SCA patients, irrespective of the specific genotype, and, if assessed, visual acuity seems to be reduced in SCA cohorts in general. Reports of retinal and optic nerve involvement are inconsistent even within the same SCA subtypes but these data are limited to small patient groups which are not homogenous regarding disease duration, disease severity, and methods of examination.

In the absence of visible morphological retinal changes in the SCA-PRKCG cohort, functional deficits might be based on ultrastructural changes and there is some support to this hypothesis: The PKCγ is only expressed in a small amount of cells in the retina and the subsequent structures of the visual pathway [13, 16] especially in cells of the magnocellular visual system [13]. Even a complete loss of these cells might not be detectable in OCT images or become apparent in between-group comparisons of retinal layer thickness. Interestingly, the magnocellular visual system is responsible for the detection of movement and contrast sensitivity, while the parvocellular system is important for color vision. This is interesting because most subscores of the NEI-VFQ were worse in the SCA-PRKCG cohort while color vision was preserved, which would be in line with an affection of the magnocellular visual pathway in SCA-PRKCG.

In the mouse cerebellum, mutant PKCγ impedes the pruning of surplus synapses leading to dysfunctional synapses and localized Purkinje cell loss [63]. Again, a similar pattern of damage in the visual pathway—dysfunctional synapses and localized atrophy—could have been missed by OCT. Of note, cross-sectional inspection of structural brain MRI in the current SCA-PRKCG cohort did not support progression of cerebellar atrophy with longer-standing disease (despite progression of ataxia symptoms), but revealed the presence of cerebellar atrophy even years before the manifestation of ataxia in one case [45]. A similar mismatch of structural findings and patient’s functions seems also possible at the level of the retina. However, given the limitations when interpreting the visual contrast sensitivity results, the hypothesis of an ultrastructural damage of the visual pathway in SCA-PRKCG is highly hypothetical and needs further evaluation. Histopathological analysis of the retina in SCA-PRKCG patients would be particularly important, e.g., to search for retinal pericellular vacuoles and pathology of PRKCγ-expressing cell types.

Finally, oculomotor deficits may serve as an explanation of reduced visual acuity and vision-related quality of life. In our opinion, difficulties in alignment are the most probable explanation, why binocular but not monocular visual acuity results are reduced in SCA-PRKCG patients compared with HC. Furthermore, oculomotor deficits serve as an explanation for the high rate of fixation loss in the visual field tests. Oculomotor deficits—at least saccadic eye movements—were seen in every patient of the SCA-PRKCG cohort. However, while abnormal VOR might reduce visual acuity during motion [64], visual acuity and contrast sensitivity were assessed in a static manner in the current study. Moreover, VOR alteration was only described in two patients and disturbed suppression of fixation was unrelated to worse contrast sensitivity results. Oscillopsia was only prominent in three patients, all of them presenting with normal contrast sensitivity and visual acuity results. Likewise, patients with moderate to severe/constant diplopia presented normal contrast sensitivity tests. In conclusion, we did not find one specific oculomotor deficit, which could explain the reduced binocular visual acuity or vision-related quality of life in PRKCG patients. Nonetheless, even if those deficits might not explain reduced visual acuity, the total impact of all oculomotor and alignment deficits (e.g., diplopia, oscillopsia) together may contribute to the reduced vision-related quality of life in SCA-PRKCG patients.

This hypothesis is corroborated by findings in other SCAs: Kedar et al. described a reduced vision-related quality of life in an ataxia cohort of 19 patients suffering from SCA-ATXN1, SCA-ATXN3, or SCA-CACNA1A with reduced scores compared with established normative data of NEI-VFQ. They also found a reduced binocular contrast sensitivity in the SCA cohort. Furthermore, patient self-report indicated disturbance of near-, distance-, and peripheral vision, as was the case in our study. The authors hypothesized that reduced vision-related quality of life in SCA may be generally caused by “ocular motility and alignment deficits” [20]. However, as opposed to study, monocular and binocular high-contrast visual acuity was unimpaired.

Reduced low-contrast sensitivity or visual acuity was described in several neurodegenerative disorders such as Parkinson’s disease, Friedreich ataxia, or even spinocerebellar ataxias [20, 65, 66]. However, in nearly all of these diseases, a concomitant affection of the afferent visual pathway was detected as a causal factor for visual acuity deficits. Rabiah et al. found reduced visual acuity results in a mixed ataxia cohort in only 16% of patients [67], which again supports that ataxia does not automatically lead to reduced visual acuity.

In sum, we conclude that reduced visual acuity results in SCA-PRKCG patients are of multifactorial nature. Technical issues, concomitant diseases (depression, cognitive impairment, reduced information processing speed), and oculomotor deficits might contribute to visual impairment.

Regarding the vision-related quality of life, we also have to take into account that this is a subjective measure which does not necessarily reflect standard visual function tests. This was seen in patients with idiopathic infantile nystagmus syndrome—as an example of an isolated oculomotor deficit syndrome—who showed reduced vision-related quality of life without concordant changes in visual acuity measurements [68]. Again, mood disorders may result in an overestimation of the visual impairment. While the differentiated self-assessment with preserved dimensions of general vision, well-being, and color vision or ocular pain argues strongly against this hypothesis, patients might misinterpret or even ignore their actual visual deficits. Especially patients with neurological deficits might underestimate their visual deficits [69].

While this study provides the first systematic description of visual disturbances in a SCA-PRKCG cohort, it has several limitations. Firstly, due to the rarity of SCA-PRKCG, only a small number of patients were included. Secondly, we ran a large number of tests in an exploratory approach; hence, significant results have to be interpreted with caution. Thirdly, we did not perform electrophysiological examinations in our patients. Electroretinography (ERG) might have helped to better detect and localize deficits in the afferent visual system of SCA-PRKCG patients. MRI and especially diffusion tensor imaging (DTI) could help to evaluate the microstructural integrity of the posterior visual pathway [70].

Finally, we describe one patient, who suffered from ataxia but carried a VUS in the catalytic domain of PRKCG (R634H). While mutations in the catalytic domain are less common than in the regulatory domain of PKCγ, the phenotype has been suggested as more complex [8]. Interestingly, this patient was the only patient in our cohort with a remarkable pathology in both fundus images, more precisely an epitheliopathy of unknown origin. The etiology of epitheliopathies comprises a wide range of differential diagnoses from age-related macular degeneration to hereditary diseases [71]. A cone-rod dystrophy is a characteristic symptom of SCA-ATXN7 [23] and involvement of the photoreceptor layer has been linked to SCA-ATXN1 [72]. In the light of unremarkable retinal findings in all confirmed SCA-PRKCG patients reported here, this may either suggest a different genotype than SCA-PRKCG for this case, which is also supported by structural brain MRI findings [45] or a specific (retinal) phenotype that has hitherto not been reported.

There is increasing evidence that patients with spinocerebellar ataxias suffer from a reduced vision-related quality of life. However, due to the diversity in pathomechanisms and systems affected in different SCA genotypes, the clinical correlate of this finding remains unclear. A larger sample size of different SCA patients will help to better segregate between oculomotor deficits and retinal changes as a putative cause of reduced VRQoL. Furthermore, as some SCAs may share a common pathomechanism as suggested for SCA-PRKCG and SCA-ATXN1 [21], elucidation of visual impairment in one of those diseases could help to better understand SCAs in general and delineate possibly common pathways that lead to visual impairment in SCAs.

For SCA-PRKCG, OCT data rather rule out retinal atrophy as a contributor to visual impairment and reduced VRQoL. Multiple factors might contribute to lower test results for binocular visual acuity and vision-related quality of life such as concomitant diseases, especially depression, cognitive impairment, and oculomotor misalignment as well as technical issues for visual acuity testing (e.g., habitual correction, selection bias). Further studies should evaluate ultrastructural changes in the visual pathway of SCA-PRKCG patients.

## Conclusion

In this pilot study, we investigated visual impairment in a cohort of SCA-PRKCG patients. While patients with SCA-PRKCG showed lower binocular, but not monocular visual acuity for high- and low-contrast and lower vision-related quality of life compared with age- and sex-matched healthy controls, we found no changes in the retinal structure on OCT. Hence, in contrast to other SCAs, we can rule out a maculopathy or retinal thinning as a cause of reduced vision-related quality of life in SCA-PRKCG patients. Concomitant diseases, oculomotor deficits, and cognitive impairment might serve as explanations for the reduced contrast sensitivity in the current cohort.

## Electronic Supplementary Material

ESM 1 (DOCX 30 kb)

Table 4 Overview of individual patient results.Individual patient results of both eyes in the fundus picture and visual acuity tests as well as ratings of ataxia severity (SARA) and disease duration. Patients are ordered according to the loci of their variant in the PRKCG gene. Abbreviations: FACT: Functional Acuity Contrast Test, logMAR: logarithm of the minimal angle of resolution, SARA: scale for the assessment and rating of ataxia, VUS: variant of unknown significance (DOCX 21 kb)

Table 5 Individual OCT results in SCA-PRKCG. Individual OCT results of both eyes from every patient. Apart from the total macular volume, all parameters represent layer thickness measurements measured in μm. Abbreviations: GCIPL: ganglion cell and inner plexiform layer, INL – inner nuclear layer, mRNFL – macular retinal nerve fibre layer, N/T ratio – nasal to temporal ratio, OCT: Optical coherence tomography, ORL: outer retinal layers from outer plexiform layer to Bruch’s membrane, PMB: papillo-macular bundle, pRNFL: peripapillary retinal nerve fibre layer, RNFL-I – inferior retinal nerve fibre layer, RNFL-N – nasal retinal nerve fibre layer, RNFL-S – superior retinal nerve fibre layer, RNFL-T – temporal retinal nerve fibre layer, TMV: total macular volume (in mm³). (DOCX 20 kb)
