# Supplementary material for: Investigation of Visual System Involvement in Spinocerebellar Ataxia Type 14
Source: Cerebellum. 2020 Apr 27;19(4):469–82. doi: 10.1007/s12311-020-01130-w (PMC7351844; doi:10.1007/s12311-020-01130-w)
Supplement: Supplementary file 1 — (DOCX 30 kb) [file 12311_2020_1130_MOESM1_ESM.docx]

1. **R-Syntax:**

“P values were calculated using SPSS as described in the statistical part of the main paper and

integrated in the title.

## Open library ggplot 2, labelling of both groups

library(ggplot2)

data$group = factor(data$group, levels = 1:2, labels = c("SCA14", "HC"))

## creating boxplots

ggplot(data=data, aes(x=data$group, y=data$health_general))+geom_boxplot()+labs (title = "A) General health p=0.001")

a1 <- ggplot(data=data, aes(x=data$group, y=data$health_general))+geom_boxplot()+labs (title = "A) General health p=0.001")

## removing redundant axis labeling

a1 + theme(axis.title = element_blank())

a2 <- a1 + theme(axis.title = element_blank())

## adding dots for every single patient to the boxplot

a2 + geom_dotplot(binaxis='y', stackdir='center', dotsize=1.5)

a3 <- a2 + geom_dotplot(binaxis='y', stackdir='center', dotsize=1.5)

## adjusting font size of the title and axes

a3 + theme (plot.title = element_text (face="bold", size = 28), axis.text.x = element_text(face="bold", vjust=1, size=28),axis.text.y = element_text (face="bold", vjust=1, size = 28))

a4 <- a3 + theme (plot.title = element_text (face="bold", size = 28), axis.text.x = element_text(face="bold", vjust=1, size=28),axis.text.y = element_text (face="bold", vjust=1, size = 28))

##The same procedure was done for all the other NEI-VFQ items

##Grouping all elements with ggarrange

library(ggpubr)

ggarrange(a4,b4,c4,d4,e4,f4,g4,h4,i4,j4,k4,l4, ncol=3, nrow=4)

##adjusting width to 1842 and height to 1448.”

1. **SPSS-Syntax:**

DATASET ACTIVATE DataSet1.

USE ALL.

COMPUTE filter_$=(statistically_eval = 1 & attribution = 1).

VARIABLE LABELS filter_$ 'statistically_eval = 1 & attribution = 1 (FILTER)'.

VALUE LABELS filter_$ 0 'Not Selected' 1 'Selected'.

FORMATS filter_$ (f1.0).

FILTER BY filter_$.

EXECUTE.

DESCRIPTIVES VARIABLES=Age SARA_score Disease_duration logMAR_exact FACT

/STATISTICS=MEAN STDDEV MIN MAX.

USE ALL.

COMPUTE filter_$=(statistically_eval = 1 & attribution = 2).

VARIABLE LABELS filter_$ 'statistically_eval = 1 & attribution = 2 (FILTER)'.

VALUE LABELS filter_$ 0 'Not Selected' 1 'Selected'.

FORMATS filter_$ (f1.0).

FILTER BY filter_$.

EXECUTE.

DESCRIPTIVES VARIABLES=Age SARA_score Disease_duration logMAR_exact FACT

/STATISTICS=MEAN STDDEV MIN MAX.

USE ALL.

COMPUTE filter_$=(statistically_eval = 1 ).

VARIABLE LABELS filter_$ 'statistically_eval = 1 (FILTER)'.

VALUE LABELS filter_$ 0 'Not Selected' 1 'Selected'.

FORMATS filter_$ (f1.0).

FILTER BY filter_$.

EXECUTE.

NPAR TESTS

/M-W= Age BY attribution(1 2)

/MISSING ANALYSIS.

CROSSTABS

/TABLES=attribution BY Gender

/FORMAT=AVALUE TABLES

/STATISTICS=CHISQ

/CELLS=COUNT

/COUNT ROUND CELL.

NPAR TESTS

/M-W= FACT logMAR_exact BY attribution(1 2)

/MISSING ANALYSIS.

ATASET ACTIVATE DataSet2.

USE ALL.

COMPUTE filter_$=(statistically_eval = 1 & attribution = 1).

VARIABLE LABELS filter_$ 'statistically_eval = 1 & attribution = 1 (FILTER)'.

VALUE LABELS filter_$ 0 'Not Selected' 1 'Selected'.

FORMATS filter_$ (f1.0).

FILTER BY filter_$.

EXECUTE.

DESCRIPTIVES VARIABLES=logMARexact

/STATISTICS=MEAN STDDEV MIN MAX.

USE ALL.

COMPUTE filter_$=(statistically_eval = 1 & attribution = 2).

VARIABLE LABELS filter_$ 'statistically_eval = 1 & attribution = 2 (FILTER)'.

VALUE LABELS filter_$ 0 'Not Selected' 1 'Selected'.

FORMATS filter_$ (f1.0).

FILTER BY filter_$.

EXECUTE.

DESCRIPTIVES VARIABLES=logMARexact

/STATISTICS=MEAN STDDEV MIN MAX.

USE ALL.

COMPUTE filter_$=(statistically_eval = 1).

VARIABLE LABELS filter_$ 'statistically_eval = 1 (FILTER)'.

VALUE LABELS filter_$ 0 'Not Selected' 1 'Selected'.

FORMATS filter_$ (f1.0).

FILTER BY filter_$.

EXECUTE.

* Generalized Estimation Equations

GENLIN logMARexact BY attribution (ORDER=ASCENDING)

/MODEL attribution INTERCEPT=YES

DISTRIBUTION=NORMAL LINK=IDENTITY

/CRITERIA SCALE=MLE PCONVERGE=1E-006(ABSOLUTE) SINGULAR=1E-012 ANALYSISTYPE=3(WALD) CILEVEL=95

LIKELIHOOD=FULL

/REPEATED SUBJECT=Eye_ID WITHINSUBJECT=Eye SORT=YES CORRTYPE=EXCHANGEABLE ADJUSTCORR=YES

COVB=ROBUST MAXITERATIONS=100 PCONVERGE=1e-006(ABSOLUTE) UPDATECORR=1

/MISSING CLASSMISSING=EXCLUDE

/PRINT CPS DESCRIPTIVES MODELINFO FIT SUMMARY SOLUTION.

DATASET ACTIVATE DataSet1.

USE ALL.

COMPUTE filter_$=(statistically_eval = 1 & attribution = 1).

VARIABLE LABELS filter_$ 'statistically_eval = 1 & attribution = 1 (FILTER)'.

VALUE LABELS filter_$ 0 'Not Selected' 1 'Selected'.

FORMATS filter_$ (f1.0).

FILTER BY filter_$.

EXECUTE.

DESCRIPTIVES VARIABLES=health_general vision_general Well_being ocular_pain near_vision

vision_distance Vision_peripheral social_function vision_color role_limitation dependency driving

tired_eyelids bright_sunlight car_parking using_a_pc different_sight eyelid_appearance blurry_fuzzy

moving_objects double_vision drooping_eyelids NOS_composite VFQ25_composite Total_composite

/STATISTICS=MEAN STDDEV MIN MAX.

USE ALL.

COMPUTE filter_$=(statistically_eval = 1 & attribution = 2).

VARIABLE LABELS filter_$ 'statistically_eval = 1 & attribution = 2 (FILTER)'.

VALUE LABELS filter_$ 0 'Not Selected' 1 'Selected'.

FORMATS filter_$ (f1.0).

FILTER BY filter_$.

EXECUTE.

DESCRIPTIVES VARIABLES=health_general vision_general Well_being ocular_pain near_vision

vision_distance Vision_peripheral social_function vision_color role_limitation dependency driving

tired_eyelids bright_sunlight car_parking using_a_pc different_sight eyelid_appearance blurry_fuzzy

moving_objects double_vision drooping_eyelids NOS_composite VFQ25_composite Total_composite

/STATISTICS=MEAN STDDEV MIN MAX.

USE ALL.

COMPUTE filter_$=(statistically_eval = 1 ).

VARIABLE LABELS filter_$ 'statistically_eval = 1 (FILTER)'.

VALUE LABELS filter_$ 0 'Not Selected' 1 'Selected'.

FORMATS filter_$ (f1.0).

FILTER BY filter_$.

EXECUTE.

NPAR TESTS

/M-W= health_general vision_general Well_being ocular_pain near_vision vision_distance

Vision_peripheral social_function vision_color role_limitation dependency driving tired_eyelids

bright_sunlight car_parking using_a_pc different_sight eyelid_appearance blurry_fuzzy

moving_objects double_vision drooping_eyelids NOS_composite VFQ25_composite Total_composite BY

attribution(1 2)

/MISSING ANALYSIS.

DATASET ACTIVATE DataSet2.

USE ALL.

COMPUTE filter_$=(statistically_eval = 1 & attribution = 1).

VARIABLE LABELS filter_$ 'statistically_eval = 1 & attribution = 1 (FILTER)'.

VALUE LABELS filter_$ 0 'Not Selected' 1 'Selected'.

FORMATS filter_$ (f1.0).

FILTER BY filter_$.

EXECUTE.

DESCRIPTIVES VARIABLES=RNFL_G RNFLMean_PMB RNFL_I RNFL_S RNFL_N RNFL_T RNFL_NT_Ratio TMVmm mRNFL

GCIPL INL ORL

/STATISTICS=MEAN STDDEV MIN MAX.

USE ALL.

COMPUTE filter_$=(statistically_eval = 1 & attribution = 2).

VARIABLE LABELS filter_$ 'statistically_eval = 1 & attribution = 2 (FILTER)'.

VALUE LABELS filter_$ 0 'Not Selected' 1 'Selected'.

FORMATS filter_$ (f1.0).

FILTER BY filter_$.

EXECUTE.

DESCRIPTIVES VARIABLES=RNFL_G RNFLMean_PMB RNFL_I RNFL_S RNFL_N RNFL_T RNFL_NT_Ratio TMVmm mRNFL

GCIPL INL ORL

/STATISTICS=MEAN STDDEV MIN MAX.

USE ALL.

COMPUTE filter_$=(statistically_eval = 1 ).

VARIABLE LABELS filter_$ 'statistically_eval = 1 (FILTER)'.

VALUE LABELS filter_$ 0 'Not Selected' 1 'Selected'.

FORMATS filter_$ (f1.0).

FILTER BY filter_$.

EXECUTE.

* Generalized Estimation Equations

GENLIN RNFL_G BY attribution (ORDER=ASCENDING)

/MODEL attribution INTERCEPT=YES

DISTRIBUTION=NORMAL LINK=IDENTITY

/CRITERIA SCALE=MLE PCONVERGE=1E-006(ABSOLUTE) SINGULAR=1E-012 ANALYSISTYPE=3(WALD) CILEVEL=95

LIKELIHOOD=FULL

/REPEATED SUBJECT=Eye_ID WITHINSUBJECT=Eye SORT=YES CORRTYPE=EXCHANGEABLE ADJUSTCORR=YES

COVB=ROBUST MAXITERATIONS=100 PCONVERGE=1e-006(ABSOLUTE) UPDATECORR=1

/MISSING CLASSMISSING=EXCLUDE

/PRINT CPS DESCRIPTIVES MODELINFO FIT SUMMARY SOLUTION.

* Generalized Estimation Equations

GENLIN RNFLMean_PMB BY attribution (ORDER=ASCENDING)

/MODEL attribution INTERCEPT=YES

DISTRIBUTION=NORMAL LINK=IDENTITY

/CRITERIA SCALE=MLE PCONVERGE=1E-006(ABSOLUTE) SINGULAR=1E-012 ANALYSISTYPE=3(WALD) CILEVEL=95

LIKELIHOOD=FULL

/REPEATED SUBJECT=Eye_ID WITHINSUBJECT=Eye SORT=YES CORRTYPE=EXCHANGEABLE ADJUSTCORR=YES

COVB=ROBUST MAXITERATIONS=100 PCONVERGE=1e-006(ABSOLUTE) UPDATECORR=1

/MISSING CLASSMISSING=EXCLUDE

/PRINT CPS DESCRIPTIVES MODELINFO FIT SUMMARY SOLUTION.

* Generalized Estimation Equations

GENLIN RNFL_I BY attribution (ORDER=ASCENDING)

/MODEL attribution INTERCEPT=YES

DISTRIBUTION=NORMAL LINK=IDENTITY

/CRITERIA SCALE=MLE PCONVERGE=1E-006(ABSOLUTE) SINGULAR=1E-012 ANALYSISTYPE=3(WALD) CILEVEL=95

LIKELIHOOD=FULL

/REPEATED SUBJECT=Eye_ID WITHINSUBJECT=Eye SORT=YES CORRTYPE=EXCHANGEABLE ADJUSTCORR=YES

COVB=ROBUST MAXITERATIONS=100 PCONVERGE=1e-006(ABSOLUTE) UPDATECORR=1

/MISSING CLASSMISSING=EXCLUDE

/PRINT CPS DESCRIPTIVES MODELINFO FIT SUMMARY SOLUTION.

* Generalized Estimation Equations

GENLIN RNFL_S BY attribution (ORDER=ASCENDING)

/MODEL attribution INTERCEPT=YES

DISTRIBUTION=NORMAL LINK=IDENTITY

/CRITERIA SCALE=MLE PCONVERGE=1E-006(ABSOLUTE) SINGULAR=1E-012 ANALYSISTYPE=3(WALD) CILEVEL=95

LIKELIHOOD=FULL

/REPEATED SUBJECT=Eye_ID WITHINSUBJECT=Eye SORT=YES CORRTYPE=EXCHANGEABLE ADJUSTCORR=YES

COVB=ROBUST MAXITERATIONS=100 PCONVERGE=1e-006(ABSOLUTE) UPDATECORR=1

/MISSING CLASSMISSING=EXCLUDE

/PRINT CPS DESCRIPTIVES MODELINFO FIT SUMMARY SOLUTION.

* Generalized Estimation Equations

GENLIN RNFL_N BY attribution (ORDER=ASCENDING)

/MODEL attribution INTERCEPT=YES

DISTRIBUTION=NORMAL LINK=IDENTITY

/CRITERIA SCALE=MLE PCONVERGE=1E-006(ABSOLUTE) SINGULAR=1E-012 ANALYSISTYPE=3(WALD) CILEVEL=95

LIKELIHOOD=FULL

/REPEATED SUBJECT=Eye_ID WITHINSUBJECT=Eye SORT=YES CORRTYPE=EXCHANGEABLE ADJUSTCORR=YES

COVB=ROBUST MAXITERATIONS=100 PCONVERGE=1e-006(ABSOLUTE) UPDATECORR=1

/MISSING CLASSMISSING=EXCLUDE

/PRINT CPS DESCRIPTIVES MODELINFO FIT SUMMARY SOLUTION.

* Generalized Estimation Equations

GENLIN RNFL_T BY attribution (ORDER=ASCENDING)

/MODEL attribution INTERCEPT=YES

DISTRIBUTION=NORMAL LINK=IDENTITY

/CRITERIA SCALE=MLE PCONVERGE=1E-006(ABSOLUTE) SINGULAR=1E-012 ANALYSISTYPE=3(WALD) CILEVEL=95

LIKELIHOOD=FULL

/REPEATED SUBJECT=Eye_ID WITHINSUBJECT=Eye SORT=YES CORRTYPE=EXCHANGEABLE ADJUSTCORR=YES

COVB=ROBUST MAXITERATIONS=100 PCONVERGE=1e-006(ABSOLUTE) UPDATECORR=1

/MISSING CLASSMISSING=EXCLUDE

/PRINT CPS DESCRIPTIVES MODELINFO FIT SUMMARY SOLUTION.

* Generalized Estimation Equations

GENLIN RNFL_NT_Ratio BY attribution (ORDER=ASCENDING)

/MODEL attribution INTERCEPT=YES

DISTRIBUTION=NORMAL LINK=IDENTITY

/CRITERIA SCALE=MLE PCONVERGE=1E-006(ABSOLUTE) SINGULAR=1E-012 ANALYSISTYPE=3(WALD) CILEVEL=95

LIKELIHOOD=FULL

/REPEATED SUBJECT=Eye_ID WITHINSUBJECT=Eye SORT=YES CORRTYPE=EXCHANGEABLE ADJUSTCORR=YES

COVB=ROBUST MAXITERATIONS=100 PCONVERGE=1e-006(ABSOLUTE) UPDATECORR=1

/MISSING CLASSMISSING=EXCLUDE

/PRINT CPS DESCRIPTIVES MODELINFO FIT SUMMARY SOLUTION.

* Generalized Estimation Equations

GENLIN TMVmm BY attribution (ORDER=ASCENDING)

/MODEL attribution INTERCEPT=YES

DISTRIBUTION=NORMAL LINK=IDENTITY

/CRITERIA SCALE=MLE PCONVERGE=1E-006(ABSOLUTE) SINGULAR=1E-012 ANALYSISTYPE=3(WALD) CILEVEL=95

LIKELIHOOD=FULL

/REPEATED SUBJECT=Eye_ID WITHINSUBJECT=Eye SORT=YES CORRTYPE=EXCHANGEABLE ADJUSTCORR=YES

COVB=ROBUST MAXITERATIONS=100 PCONVERGE=1e-006(ABSOLUTE) UPDATECORR=1

/MISSING CLASSMISSING=EXCLUDE

/PRINT CPS DESCRIPTIVES MODELINFO FIT SUMMARY SOLUTION.

* Generalized Estimation Equations

GENLIN mRNFL BY attribution (ORDER=ASCENDING)

/MODEL attribution INTERCEPT=YES

DISTRIBUTION=NORMAL LINK=IDENTITY

/CRITERIA SCALE=MLE PCONVERGE=1E-006(ABSOLUTE) SINGULAR=1E-012 ANALYSISTYPE=3(WALD) CILEVEL=95

LIKELIHOOD=FULL

/REPEATED SUBJECT=Eye_ID WITHINSUBJECT=Eye SORT=YES CORRTYPE=EXCHANGEABLE ADJUSTCORR=YES

COVB=ROBUST MAXITERATIONS=100 PCONVERGE=1e-006(ABSOLUTE) UPDATECORR=1

/MISSING CLASSMISSING=EXCLUDE

/PRINT CPS DESCRIPTIVES MODELINFO FIT SUMMARY SOLUTION.

* Generalized Estimation Equations

GENLIN GCIPL BY attribution (ORDER=ASCENDING)

/MODEL attribution INTERCEPT=YES

DISTRIBUTION=NORMAL LINK=IDENTITY

/CRITERIA SCALE=MLE PCONVERGE=1E-006(ABSOLUTE) SINGULAR=1E-012 ANALYSISTYPE=3(WALD) CILEVEL=95

LIKELIHOOD=FULL

/REPEATED SUBJECT=Eye_ID WITHINSUBJECT=Eye SORT=YES CORRTYPE=EXCHANGEABLE ADJUSTCORR=YES

COVB=ROBUST MAXITERATIONS=100 PCONVERGE=1e-006(ABSOLUTE) UPDATECORR=1

/MISSING CLASSMISSING=EXCLUDE

/PRINT CPS DESCRIPTIVES MODELINFO FIT SUMMARY SOLUTION.

* Generalized Estimation Equations

GENLIN INL BY attribution (ORDER=ASCENDING)

/MODEL attribution INTERCEPT=YES

DISTRIBUTION=NORMAL LINK=IDENTITY

/CRITERIA SCALE=MLE PCONVERGE=1E-006(ABSOLUTE) SINGULAR=1E-012 ANALYSISTYPE=3(WALD) CILEVEL=95

LIKELIHOOD=FULL

/REPEATED SUBJECT=Eye_ID WITHINSUBJECT=Eye SORT=YES CORRTYPE=EXCHANGEABLE ADJUSTCORR=YES

COVB=ROBUST MAXITERATIONS=100 PCONVERGE=1e-006(ABSOLUTE) UPDATECORR=1

/MISSING CLASSMISSING=EXCLUDE

/PRINT CPS DESCRIPTIVES MODELINFO FIT SUMMARY SOLUTION.

* Generalized Estimation Equations

GENLIN ORL BY attribution (ORDER=ASCENDING)

/MODEL attribution INTERCEPT=YES

DISTRIBUTION=NORMAL LINK=IDENTITY

/CRITERIA SCALE=MLE PCONVERGE=1E-006(ABSOLUTE) SINGULAR=1E-012 ANALYSISTYPE=3(WALD) CILEVEL=95

LIKELIHOOD=FULL

/REPEATED SUBJECT=Eye_ID WITHINSUBJECT=Eye SORT=YES CORRTYPE=EXCHANGEABLE ADJUSTCORR=YES

COVB=ROBUST MAXITERATIONS=100 PCONVERGE=1e-006(ABSOLUTE) UPDATECORR=1

/MISSING CLASSMISSING=EXCLUDE

/PRINT CPS DESCRIPTIVES MODELINFO FIT SUMMARY SOLUTION.

DATASET ACTIVATE DataSet1.

USE ALL.

COMPUTE filter_$=(statistically_eval = 1 & attribution = 1).

VARIABLE LABELS filter_$ 'statistically_eval = 1 & attribution = 1 (FILTER)'.

VALUE LABELS filter_$ 0 'Not Selected' 1 'Selected'.

FORMATS filter_$ (f1.0).

FILTER BY filter_$.

EXECUTE.

NONPAR CORR

/VARIABLES=Age health_general vision_general Well_being ocular_pain near_vision vision_distance

Vision_peripheral social_function vision_color role_limitation dependency driving tired_eyelids

bright_sunlight car_parking using_a_pc different_sight eyelid_appearance blurry_fuzzy

moving_objects double_vision drooping_eyelids NOS_composite VFQ25_composite Total_composite

SARA_score FACT logMAR_exact Disease_duration

/PRINT=SPEARMAN TWOTAIL NOSIG

/MISSING=PAIRWISE.

DATASET ACTIVATE DataSet2.

USE ALL.

COMPUTE filter_$=(statistically_eval = 1 & attribution = 1).

VARIABLE LABELS filter_$ 'statistically_eval = 1 & attribution = 1 (FILTER)'.

VALUE LABELS filter_$ 0 'Not Selected' 1 'Selected'.

FORMATS filter_$ (f1.0).

FILTER BY filter_$.

EXECUTE.

* Generalized Estimation Equations

GENLIN RNFL_G WITH disease_duration

/MODEL disease_duration INTERCEPT=YES

DISTRIBUTION=NORMAL LINK=IDENTITY

/CRITERIA SCALE=MLE PCONVERGE=1E-006(ABSOLUTE) SINGULAR=1E-012 ANALYSISTYPE=3(WALD) CILEVEL=95

LIKELIHOOD=FULL

/REPEATED SUBJECT=Eye_ID WITHINSUBJECT=Eye SORT=YES CORRTYPE=EXCHANGEABLE ADJUSTCORR=YES

COVB=ROBUST MAXITERATIONS=100 PCONVERGE=1e-006(ABSOLUTE) UPDATECORR=1

/MISSING CLASSMISSING=EXCLUDE

/PRINT CPS DESCRIPTIVES MODELINFO FIT SUMMARY SOLUTION.

* Generalized Estimation Equations

GENLIN RNFLMean_PMB WITH disease_duration

/MODEL disease_duration INTERCEPT=YES

DISTRIBUTION=NORMAL LINK=IDENTITY

/CRITERIA SCALE=MLE PCONVERGE=1E-006(ABSOLUTE) SINGULAR=1E-012 ANALYSISTYPE=3(WALD) CILEVEL=95

LIKELIHOOD=FULL

/REPEATED SUBJECT=Eye_ID WITHINSUBJECT=Eye SORT=YES CORRTYPE=EXCHANGEABLE ADJUSTCORR=YES

COVB=ROBUST MAXITERATIONS=100 PCONVERGE=1e-006(ABSOLUTE) UPDATECORR=1

/MISSING CLASSMISSING=EXCLUDE

/PRINT CPS DESCRIPTIVES MODELINFO FIT SUMMARY SOLUTION.

* Generalized Estimation Equations

GENLIN RNFL_I WITH disease_duration

/MODEL disease_duration INTERCEPT=YES

DISTRIBUTION=NORMAL LINK=IDENTITY

/CRITERIA SCALE=MLE PCONVERGE=1E-006(ABSOLUTE) SINGULAR=1E-012 ANALYSISTYPE=3(WALD) CILEVEL=95

LIKELIHOOD=FULL

/REPEATED SUBJECT=Eye_ID WITHINSUBJECT=Eye SORT=YES CORRTYPE=EXCHANGEABLE ADJUSTCORR=YES

COVB=ROBUST MAXITERATIONS=100 PCONVERGE=1e-006(ABSOLUTE) UPDATECORR=1

/MISSING CLASSMISSING=EXCLUDE

/PRINT CPS DESCRIPTIVES MODELINFO FIT SUMMARY SOLUTION.

* Generalized Estimation Equations

GENLIN RNFL_S WITH disease_duration

/MODEL disease_duration INTERCEPT=YES

DISTRIBUTION=NORMAL LINK=IDENTITY

/CRITERIA SCALE=MLE PCONVERGE=1E-006(ABSOLUTE) SINGULAR=1E-012 ANALYSISTYPE=3(WALD) CILEVEL=95

LIKELIHOOD=FULL

/REPEATED SUBJECT=Eye_ID WITHINSUBJECT=Eye SORT=YES CORRTYPE=EXCHANGEABLE ADJUSTCORR=YES

COVB=ROBUST MAXITERATIONS=100 PCONVERGE=1e-006(ABSOLUTE) UPDATECORR=1

/MISSING CLASSMISSING=EXCLUDE

/PRINT CPS DESCRIPTIVES MODELINFO FIT SUMMARY SOLUTION.

* Generalized Estimation Equations

GENLIN RNFL_N WITH disease_duration

/MODEL disease_duration INTERCEPT=YES

DISTRIBUTION=NORMAL LINK=IDENTITY

/CRITERIA SCALE=MLE PCONVERGE=1E-006(ABSOLUTE) SINGULAR=1E-012 ANALYSISTYPE=3(WALD) CILEVEL=95

LIKELIHOOD=FULL

/REPEATED SUBJECT=Eye_ID WITHINSUBJECT=Eye SORT=YES CORRTYPE=EXCHANGEABLE ADJUSTCORR=YES

COVB=ROBUST MAXITERATIONS=100 PCONVERGE=1e-006(ABSOLUTE) UPDATECORR=1

/MISSING CLASSMISSING=EXCLUDE

/PRINT CPS DESCRIPTIVES MODELINFO FIT SUMMARY SOLUTION.

* Generalized Estimation Equations

GENLIN RNFL_T WITH disease_duration

/MODEL disease_duration INTERCEPT=YES

DISTRIBUTION=NORMAL LINK=IDENTITY

/CRITERIA SCALE=MLE PCONVERGE=1E-006(ABSOLUTE) SINGULAR=1E-012 ANALYSISTYPE=3(WALD) CILEVEL=95

LIKELIHOOD=FULL

/REPEATED SUBJECT=Eye_ID WITHINSUBJECT=Eye SORT=YES CORRTYPE=EXCHANGEABLE ADJUSTCORR=YES

COVB=ROBUST MAXITERATIONS=100 PCONVERGE=1e-006(ABSOLUTE) UPDATECORR=1

/MISSING CLASSMISSING=EXCLUDE

/PRINT CPS DESCRIPTIVES MODELINFO FIT SUMMARY SOLUTION.

* Generalized Estimation Equations

GENLIN RNFL_NT_Ratio WITH disease_duration

/MODEL disease_duration INTERCEPT=YES

DISTRIBUTION=NORMAL LINK=IDENTITY

/CRITERIA SCALE=MLE PCONVERGE=1E-006(ABSOLUTE) SINGULAR=1E-012 ANALYSISTYPE=3(WALD) CILEVEL=95

LIKELIHOOD=FULL

/REPEATED SUBJECT=Eye_ID WITHINSUBJECT=Eye SORT=YES CORRTYPE=EXCHANGEABLE ADJUSTCORR=YES

COVB=ROBUST MAXITERATIONS=100 PCONVERGE=1e-006(ABSOLUTE) UPDATECORR=1

/MISSING CLASSMISSING=EXCLUDE

/PRINT CPS DESCRIPTIVES MODELINFO FIT SUMMARY SOLUTION.

* Generalized Estimation Equations

GENLIN TMVmm WITH disease_duration

/MODEL disease_duration INTERCEPT=YES

DISTRIBUTION=NORMAL LINK=IDENTITY

/CRITERIA SCALE=MLE PCONVERGE=1E-006(ABSOLUTE) SINGULAR=1E-012 ANALYSISTYPE=3(WALD) CILEVEL=95

LIKELIHOOD=FULL

/REPEATED SUBJECT=Eye_ID WITHINSUBJECT=Eye SORT=YES CORRTYPE=EXCHANGEABLE ADJUSTCORR=YES

COVB=ROBUST MAXITERATIONS=100 PCONVERGE=1e-006(ABSOLUTE) UPDATECORR=1

/MISSING CLASSMISSING=EXCLUDE

/PRINT CPS DESCRIPTIVES MODELINFO FIT SUMMARY SOLUTION.

* Generalized Estimation Equations

GENLIN mRNFL WITH disease_duration

/MODEL disease_duration INTERCEPT=YES

DISTRIBUTION=NORMAL LINK=IDENTITY

/CRITERIA SCALE=MLE PCONVERGE=1E-006(ABSOLUTE) SINGULAR=1E-012 ANALYSISTYPE=3(WALD) CILEVEL=95

LIKELIHOOD=FULL

/REPEATED SUBJECT=Eye_ID WITHINSUBJECT=Eye SORT=YES CORRTYPE=EXCHANGEABLE ADJUSTCORR=YES

COVB=ROBUST MAXITERATIONS=100 PCONVERGE=1e-006(ABSOLUTE) UPDATECORR=1

/MISSING CLASSMISSING=EXCLUDE

/PRINT CPS DESCRIPTIVES MODELINFO FIT SUMMARY SOLUTION.

* Generalized Estimation Equations

GENLIN GCIPL WITH disease_duration

/MODEL disease_duration INTERCEPT=YES

DISTRIBUTION=NORMAL LINK=IDENTITY

/CRITERIA SCALE=MLE PCONVERGE=1E-006(ABSOLUTE) SINGULAR=1E-012 ANALYSISTYPE=3(WALD) CILEVEL=95

LIKELIHOOD=FULL

/REPEATED SUBJECT=Eye_ID WITHINSUBJECT=Eye SORT=YES CORRTYPE=EXCHANGEABLE ADJUSTCORR=YES

COVB=ROBUST MAXITERATIONS=100 PCONVERGE=1e-006(ABSOLUTE) UPDATECORR=1

/MISSING CLASSMISSING=EXCLUDE

/PRINT CPS DESCRIPTIVES MODELINFO FIT SUMMARY SOLUTION.

* Generalized Estimation Equations

GENLIN INL WITH disease_duration

/MODEL disease_duration INTERCEPT=YES

DISTRIBUTION=NORMAL LINK=IDENTITY

/CRITERIA SCALE=MLE PCONVERGE=1E-006(ABSOLUTE) SINGULAR=1E-012 ANALYSISTYPE=3(WALD) CILEVEL=95

LIKELIHOOD=FULL

/REPEATED SUBJECT=Eye_ID WITHINSUBJECT=Eye SORT=YES CORRTYPE=EXCHANGEABLE ADJUSTCORR=YES

COVB=ROBUST MAXITERATIONS=100 PCONVERGE=1e-006(ABSOLUTE) UPDATECORR=1

/MISSING CLASSMISSING=EXCLUDE

/PRINT CPS DESCRIPTIVES MODELINFO FIT SUMMARY SOLUTION.

* Generalized Estimation Equations

GENLIN ORL WITH disease_duration

/MODEL disease_duration INTERCEPT=YES

DISTRIBUTION=NORMAL LINK=IDENTITY

/CRITERIA SCALE=MLE PCONVERGE=1E-006(ABSOLUTE) SINGULAR=1E-012 ANALYSISTYPE=3(WALD) CILEVEL=95

LIKELIHOOD=FULL

/REPEATED SUBJECT=Eye_ID WITHINSUBJECT=Eye SORT=YES CORRTYPE=EXCHANGEABLE ADJUSTCORR=YES

COVB=ROBUST MAXITERATIONS=100 PCONVERGE=1e-006(ABSOLUTE) UPDATECORR=1

/MISSING CLASSMISSING=EXCLUDE

/PRINT CPS DESCRIPTIVES MODELINFO FIT SUMMARY SOLUTION.

* Generalized Estimation Equations

GENLIN RNFL_G WITH SARA_score

/MODEL SARA_score INTERCEPT=YES

DISTRIBUTION=NORMAL LINK=IDENTITY

/CRITERIA SCALE=MLE PCONVERGE=1E-006(ABSOLUTE) SINGULAR=1E-012 ANALYSISTYPE=3(WALD) CILEVEL=95

LIKELIHOOD=FULL

/REPEATED SUBJECT=Eye_ID WITHINSUBJECT=Eye SORT=YES CORRTYPE=EXCHANGEABLE ADJUSTCORR=YES

COVB=ROBUST MAXITERATIONS=100 PCONVERGE=1e-006(ABSOLUTE) UPDATECORR=1

/MISSING CLASSMISSING=EXCLUDE

/PRINT CPS DESCRIPTIVES MODELINFO FIT SUMMARY SOLUTION.

* Generalized Estimation Equations

GENLIN RNFLMean_PMB WITH SARA_score

/MODEL SARA_score INTERCEPT=YES

DISTRIBUTION=NORMAL LINK=IDENTITY

/CRITERIA SCALE=MLE PCONVERGE=1E-006(ABSOLUTE) SINGULAR=1E-012 ANALYSISTYPE=3(WALD) CILEVEL=95

LIKELIHOOD=FULL

/REPEATED SUBJECT=Eye_ID WITHINSUBJECT=Eye SORT=YES CORRTYPE=EXCHANGEABLE ADJUSTCORR=YES

COVB=ROBUST MAXITERATIONS=100 PCONVERGE=1e-006(ABSOLUTE) UPDATECORR=1

/MISSING CLASSMISSING=EXCLUDE

/PRINT CPS DESCRIPTIVES MODELINFO FIT SUMMARY SOLUTION.

* Generalized Estimation Equations

GENLIN RNFL_I WITH SARA_score

/MODEL SARA_score INTERCEPT=YES

DISTRIBUTION=NORMAL LINK=IDENTITY

/CRITERIA SCALE=MLE PCONVERGE=1E-006(ABSOLUTE) SINGULAR=1E-012 ANALYSISTYPE=3(WALD) CILEVEL=95

LIKELIHOOD=FULL

/REPEATED SUBJECT=Eye_ID WITHINSUBJECT=Eye SORT=YES CORRTYPE=EXCHANGEABLE ADJUSTCORR=YES

COVB=ROBUST MAXITERATIONS=100 PCONVERGE=1e-006(ABSOLUTE) UPDATECORR=1

/MISSING CLASSMISSING=EXCLUDE

/PRINT CPS DESCRIPTIVES MODELINFO FIT SUMMARY SOLUTION.

* Generalized Estimation Equations

GENLIN RNFL_S WITH SARA_score

/MODEL SARA_score INTERCEPT=YES

DISTRIBUTION=NORMAL LINK=IDENTITY

/CRITERIA SCALE=MLE PCONVERGE=1E-006(ABSOLUTE) SINGULAR=1E-012 ANALYSISTYPE=3(WALD) CILEVEL=95

LIKELIHOOD=FULL

/REPEATED SUBJECT=Eye_ID WITHINSUBJECT=Eye SORT=YES CORRTYPE=EXCHANGEABLE ADJUSTCORR=YES

COVB=ROBUST MAXITERATIONS=100 PCONVERGE=1e-006(ABSOLUTE) UPDATECORR=1

/MISSING CLASSMISSING=EXCLUDE

/PRINT CPS DESCRIPTIVES MODELINFO FIT SUMMARY SOLUTION.

* Generalized Estimation Equations

GENLIN RNFL_N WITH SARA_score

/MODEL SARA_score INTERCEPT=YES

DISTRIBUTION=NORMAL LINK=IDENTITY

/CRITERIA SCALE=MLE PCONVERGE=1E-006(ABSOLUTE) SINGULAR=1E-012 ANALYSISTYPE=3(WALD) CILEVEL=95

LIKELIHOOD=FULL

/REPEATED SUBJECT=Eye_ID WITHINSUBJECT=Eye SORT=YES CORRTYPE=EXCHANGEABLE ADJUSTCORR=YES

COVB=ROBUST MAXITERATIONS=100 PCONVERGE=1e-006(ABSOLUTE) UPDATECORR=1

/MISSING CLASSMISSING=EXCLUDE

/PRINT CPS DESCRIPTIVES MODELINFO FIT SUMMARY SOLUTION.

* Generalized Estimation Equations

GENLIN RNFL_T WITH SARA_score

/MODEL SARA_score INTERCEPT=YES

DISTRIBUTION=NORMAL LINK=IDENTITY

/CRITERIA SCALE=MLE PCONVERGE=1E-006(ABSOLUTE) SINGULAR=1E-012 ANALYSISTYPE=3(WALD) CILEVEL=95

LIKELIHOOD=FULL

/REPEATED SUBJECT=Eye_ID WITHINSUBJECT=Eye SORT=YES CORRTYPE=EXCHANGEABLE ADJUSTCORR=YES

COVB=ROBUST MAXITERATIONS=100 PCONVERGE=1e-006(ABSOLUTE) UPDATECORR=1

/MISSING CLASSMISSING=EXCLUDE

/PRINT CPS DESCRIPTIVES MODELINFO FIT SUMMARY SOLUTION.

* Generalized Estimation Equations

GENLIN RNFL_NT_Ratio WITH SARA_score

/MODEL SARA_score INTERCEPT=YES

DISTRIBUTION=NORMAL LINK=IDENTITY

/CRITERIA SCALE=MLE PCONVERGE=1E-006(ABSOLUTE) SINGULAR=1E-012 ANALYSISTYPE=3(WALD) CILEVEL=95

LIKELIHOOD=FULL

/REPEATED SUBJECT=Eye_ID WITHINSUBJECT=Eye SORT=YES CORRTYPE=EXCHANGEABLE ADJUSTCORR=YES

COVB=ROBUST MAXITERATIONS=100 PCONVERGE=1e-006(ABSOLUTE) UPDATECORR=1

/MISSING CLASSMISSING=EXCLUDE

/PRINT CPS DESCRIPTIVES MODELINFO FIT SUMMARY SOLUTION.

* Generalized Estimation Equations

GENLIN TMVmm WITH SARA_score

/MODEL SARA_score INTERCEPT=YES

DISTRIBUTION=NORMAL LINK=IDENTITY

/CRITERIA SCALE=MLE PCONVERGE=1E-006(ABSOLUTE) SINGULAR=1E-012 ANALYSISTYPE=3(WALD) CILEVEL=95

LIKELIHOOD=FULL

/REPEATED SUBJECT=Eye_ID WITHINSUBJECT=Eye SORT=YES CORRTYPE=EXCHANGEABLE ADJUSTCORR=YES

COVB=ROBUST MAXITERATIONS=100 PCONVERGE=1e-006(ABSOLUTE) UPDATECORR=1

/MISSING CLASSMISSING=EXCLUDE

/PRINT CPS DESCRIPTIVES MODELINFO FIT SUMMARY SOLUTION.

* Generalized Estimation Equations

GENLIN mRNFL WITH SARA_score

/MODEL SARA_score INTERCEPT=YES

DISTRIBUTION=NORMAL LINK=IDENTITY

/CRITERIA SCALE=MLE PCONVERGE=1E-006(ABSOLUTE) SINGULAR=1E-012 ANALYSISTYPE=3(WALD) CILEVEL=95

LIKELIHOOD=FULL

/REPEATED SUBJECT=Eye_ID WITHINSUBJECT=Eye SORT=YES CORRTYPE=EXCHANGEABLE ADJUSTCORR=YES

COVB=ROBUST MAXITERATIONS=100 PCONVERGE=1e-006(ABSOLUTE) UPDATECORR=1

/MISSING CLASSMISSING=EXCLUDE

/PRINT CPS DESCRIPTIVES MODELINFO FIT SUMMARY SOLUTION.

* Generalized Estimation Equations

GENLIN GCIPL WITH SARA_score

/MODEL SARA_score INTERCEPT=YES

DISTRIBUTION=NORMAL LINK=IDENTITY

/CRITERIA SCALE=MLE PCONVERGE=1E-006(ABSOLUTE) SINGULAR=1E-012 ANALYSISTYPE=3(WALD) CILEVEL=95

LIKELIHOOD=FULL

/REPEATED SUBJECT=Eye_ID WITHINSUBJECT=Eye SORT=YES CORRTYPE=EXCHANGEABLE ADJUSTCORR=YES

COVB=ROBUST MAXITERATIONS=100 PCONVERGE=1e-006(ABSOLUTE) UPDATECORR=1

/MISSING CLASSMISSING=EXCLUDE

/PRINT CPS DESCRIPTIVES MODELINFO FIT SUMMARY SOLUTION.

* Generalized Estimation Equations

GENLIN INL WITH SARA_score

/MODEL SARA_score INTERCEPT=YES

DISTRIBUTION=NORMAL LINK=IDENTITY

/CRITERIA SCALE=MLE PCONVERGE=1E-006(ABSOLUTE) SINGULAR=1E-012 ANALYSISTYPE=3(WALD) CILEVEL=95

LIKELIHOOD=FULL

/REPEATED SUBJECT=Eye_ID WITHINSUBJECT=Eye SORT=YES CORRTYPE=EXCHANGEABLE ADJUSTCORR=YES

COVB=ROBUST MAXITERATIONS=100 PCONVERGE=1e-006(ABSOLUTE) UPDATECORR=1

/MISSING CLASSMISSING=EXCLUDE

/PRINT CPS DESCRIPTIVES MODELINFO FIT SUMMARY SOLUTION.

* Generalized Estimation Equations

GENLIN ORL WITH SARA_score

/MODEL SARA_score INTERCEPT=YES

DISTRIBUTION=NORMAL LINK=IDENTITY

/CRITERIA SCALE=MLE PCONVERGE=1E-006(ABSOLUTE) SINGULAR=1E-012 ANALYSISTYPE=3(WALD) CILEVEL=95

LIKELIHOOD=FULL

/REPEATED SUBJECT=Eye_ID WITHINSUBJECT=Eye SORT=YES CORRTYPE=EXCHANGEABLE ADJUSTCORR=YES

COVB=ROBUST MAXITERATIONS=100 PCONVERGE=1e-006(ABSOLUTE) UPDATECORR=1

/MISSING CLASSMISSING=EXCLUDE

/PRINT CPS DESCRIPTIVES MODELINFO FIT SUMMARY SOLUTION.

* Generalized Estimation Equations

GENLIN RNFL_G WITH Age

/MODEL Age INTERCEPT=YES

DISTRIBUTION=NORMAL LINK=IDENTITY

/CRITERIA SCALE=MLE PCONVERGE=1E-006(ABSOLUTE) SINGULAR=1E-012 ANALYSISTYPE=3(WALD) CILEVEL=95

LIKELIHOOD=FULL

/REPEATED SUBJECT=Eye_ID WITHINSUBJECT=Eye SORT=YES CORRTYPE=EXCHANGEABLE ADJUSTCORR=YES

COVB=ROBUST MAXITERATIONS=100 PCONVERGE=1e-006(ABSOLUTE) UPDATECORR=1

/MISSING CLASSMISSING=EXCLUDE

/PRINT CPS DESCRIPTIVES MODELINFO FIT SUMMARY SOLUTION.

* Generalized Estimation Equations

GENLIN RNFLMean_PMB WITH Age

/MODEL Age INTERCEPT=YES

DISTRIBUTION=NORMAL LINK=IDENTITY

/CRITERIA SCALE=MLE PCONVERGE=1E-006(ABSOLUTE) SINGULAR=1E-012 ANALYSISTYPE=3(WALD) CILEVEL=95

LIKELIHOOD=FULL

/REPEATED SUBJECT=Eye_ID WITHINSUBJECT=Eye SORT=YES CORRTYPE=EXCHANGEABLE ADJUSTCORR=YES

COVB=ROBUST MAXITERATIONS=100 PCONVERGE=1e-006(ABSOLUTE) UPDATECORR=1

/MISSING CLASSMISSING=EXCLUDE

/PRINT CPS DESCRIPTIVES MODELINFO FIT SUMMARY SOLUTION.

* Generalized Estimation Equations

GENLIN RNFL_I WITH Age

/MODEL Age INTERCEPT=YES

DISTRIBUTION=NORMAL LINK=IDENTITY

/CRITERIA SCALE=MLE PCONVERGE=1E-006(ABSOLUTE) SINGULAR=1E-012 ANALYSISTYPE=3(WALD) CILEVEL=95

LIKELIHOOD=FULL

/REPEATED SUBJECT=Eye_ID WITHINSUBJECT=Eye SORT=YES CORRTYPE=EXCHANGEABLE ADJUSTCORR=YES

COVB=ROBUST MAXITERATIONS=100 PCONVERGE=1e-006(ABSOLUTE) UPDATECORR=1

/MISSING CLASSMISSING=EXCLUDE

/PRINT CPS DESCRIPTIVES MODELINFO FIT SUMMARY SOLUTION.

* Generalized Estimation Equations

GENLIN RNFL_S WITH Age

/MODEL Age INTERCEPT=YES

DISTRIBUTION=NORMAL LINK=IDENTITY

/CRITERIA SCALE=MLE PCONVERGE=1E-006(ABSOLUTE) SINGULAR=1E-012 ANALYSISTYPE=3(WALD) CILEVEL=95

LIKELIHOOD=FULL

/REPEATED SUBJECT=Eye_ID WITHINSUBJECT=Eye SORT=YES CORRTYPE=EXCHANGEABLE ADJUSTCORR=YES

COVB=ROBUST MAXITERATIONS=100 PCONVERGE=1e-006(ABSOLUTE) UPDATECORR=1

/MISSING CLASSMISSING=EXCLUDE

/PRINT CPS DESCRIPTIVES MODELINFO FIT SUMMARY SOLUTION.

* Generalized Estimation Equations

GENLIN RNFL_N WITH Age

/MODEL Age INTERCEPT=YES

DISTRIBUTION=NORMAL LINK=IDENTITY

/CRITERIA SCALE=MLE PCONVERGE=1E-006(ABSOLUTE) SINGULAR=1E-012 ANALYSISTYPE=3(WALD) CILEVEL=95

LIKELIHOOD=FULL

/REPEATED SUBJECT=Eye_ID WITHINSUBJECT=Eye SORT=YES CORRTYPE=EXCHANGEABLE ADJUSTCORR=YES

COVB=ROBUST MAXITERATIONS=100 PCONVERGE=1e-006(ABSOLUTE) UPDATECORR=1

/MISSING CLASSMISSING=EXCLUDE

/PRINT CPS DESCRIPTIVES MODELINFO FIT SUMMARY SOLUTION.

* Generalized Estimation Equations

GENLIN RNFL_T WITH Age

/MODEL Age INTERCEPT=YES

DISTRIBUTION=NORMAL LINK=IDENTITY

/CRITERIA SCALE=MLE PCONVERGE=1E-006(ABSOLUTE) SINGULAR=1E-012 ANALYSISTYPE=3(WALD) CILEVEL=95

LIKELIHOOD=FULL

/REPEATED SUBJECT=Eye_ID WITHINSUBJECT=Eye SORT=YES CORRTYPE=EXCHANGEABLE ADJUSTCORR=YES

COVB=ROBUST MAXITERATIONS=100 PCONVERGE=1e-006(ABSOLUTE) UPDATECORR=1

/MISSING CLASSMISSING=EXCLUDE

/PRINT CPS DESCRIPTIVES MODELINFO FIT SUMMARY SOLUTION.

* Generalized Estimation Equations

GENLIN RNFL_NT_Ratio WITH Age

/MODEL Age INTERCEPT=YES

DISTRIBUTION=NORMAL LINK=IDENTITY

/CRITERIA SCALE=MLE PCONVERGE=1E-006(ABSOLUTE) SINGULAR=1E-012 ANALYSISTYPE=3(WALD) CILEVEL=95

LIKELIHOOD=FULL

/REPEATED SUBJECT=Eye_ID WITHINSUBJECT=Eye SORT=YES CORRTYPE=EXCHANGEABLE ADJUSTCORR=YES

COVB=ROBUST MAXITERATIONS=100 PCONVERGE=1e-006(ABSOLUTE) UPDATECORR=1

/MISSING CLASSMISSING=EXCLUDE

/PRINT CPS DESCRIPTIVES MODELINFO FIT SUMMARY SOLUTION.

* Generalized Estimation Equations

GENLIN TMVmm WITH Age

/MODEL Age INTERCEPT=YES

DISTRIBUTION=NORMAL LINK=IDENTITY

/CRITERIA SCALE=MLE PCONVERGE=1E-006(ABSOLUTE) SINGULAR=1E-012 ANALYSISTYPE=3(WALD) CILEVEL=95

LIKELIHOOD=FULL

/REPEATED SUBJECT=Eye_ID WITHINSUBJECT=Eye SORT=YES CORRTYPE=EXCHANGEABLE ADJUSTCORR=YES

COVB=ROBUST MAXITERATIONS=100 PCONVERGE=1e-006(ABSOLUTE) UPDATECORR=1

/MISSING CLASSMISSING=EXCLUDE

/PRINT CPS DESCRIPTIVES MODELINFO FIT SUMMARY SOLUTION.

* Generalized Estimation Equations

GENLIN mRNFL WITH Age

/MODEL Age INTERCEPT=YES

DISTRIBUTION=NORMAL LINK=IDENTITY

/CRITERIA SCALE=MLE PCONVERGE=1E-006(ABSOLUTE) SINGULAR=1E-012 ANALYSISTYPE=3(WALD) CILEVEL=95

LIKELIHOOD=FULL

/REPEATED SUBJECT=Eye_ID WITHINSUBJECT=Eye SORT=YES CORRTYPE=EXCHANGEABLE ADJUSTCORR=YES

COVB=ROBUST MAXITERATIONS=100 PCONVERGE=1e-006(ABSOLUTE) UPDATECORR=1

/MISSING CLASSMISSING=EXCLUDE

/PRINT CPS DESCRIPTIVES MODELINFO FIT SUMMARY SOLUTION.

* Generalized Estimation Equations

GENLIN GCIPL WITH Age

/MODEL Age INTERCEPT=YES

DISTRIBUTION=NORMAL LINK=IDENTITY

/CRITERIA SCALE=MLE PCONVERGE=1E-006(ABSOLUTE) SINGULAR=1E-012 ANALYSISTYPE=3(WALD) CILEVEL=95

LIKELIHOOD=FULL

/REPEATED SUBJECT=Eye_ID WITHINSUBJECT=Eye SORT=YES CORRTYPE=EXCHANGEABLE ADJUSTCORR=YES

COVB=ROBUST MAXITERATIONS=100 PCONVERGE=1e-006(ABSOLUTE) UPDATECORR=1

/MISSING CLASSMISSING=EXCLUDE

/PRINT CPS DESCRIPTIVES MODELINFO FIT SUMMARY SOLUTION.

* Generalized Estimation Equations

GENLIN INL WITH Age

/MODEL Age INTERCEPT=YES

DISTRIBUTION=NORMAL LINK=IDENTITY

/CRITERIA SCALE=MLE PCONVERGE=1E-006(ABSOLUTE) SINGULAR=1E-012 ANALYSISTYPE=3(WALD) CILEVEL=95

LIKELIHOOD=FULL

/REPEATED SUBJECT=Eye_ID WITHINSUBJECT=Eye SORT=YES CORRTYPE=EXCHANGEABLE ADJUSTCORR=YES

COVB=ROBUST MAXITERATIONS=100 PCONVERGE=1e-006(ABSOLUTE) UPDATECORR=1

/MISSING CLASSMISSING=EXCLUDE

/PRINT CPS DESCRIPTIVES MODELINFO FIT SUMMARY SOLUTION.

* Generalized Estimation Equations

GENLIN ORL WITH Age

/MODEL Age INTERCEPT=YES

DISTRIBUTION=NORMAL LINK=IDENTITY

/CRITERIA SCALE=MLE PCONVERGE=1E-006(ABSOLUTE) SINGULAR=1E-012 ANALYSISTYPE=3(WALD) CILEVEL=95

LIKELIHOOD=FULL

/REPEATED SUBJECT=Eye_ID WITHINSUBJECT=Eye SORT=YES CORRTYPE=EXCHANGEABLE ADJUSTCORR=YES

COVB=ROBUST MAXITERATIONS=100 PCONVERGE=1e-006(ABSOLUTE) UPDATECORR=1

/MISSING CLASSMISSING=EXCLUDE

/PRINT CPS DESCRIPTIVES MODELINFO FIT SUMMARY SOLUTION.
