# Supplementary material for: Investigation of Visual System Involvement in Spinocerebellar Ataxia Type 14
Source: Cerebellum. 2020 Apr 27;19(4):469–82. doi: 10.1007/s12311-020-01130-w (PMC7351844; doi:10.1007/s12311-020-01130-w)
Supplement: Supplementary file 3 — Individual OCT results in SCA-PRKCG. Individual OCT results of both eyes from every patient. Apart from the total macular volume, all parameters represent layer thickness measurements measured in μm. Abbreviations: GCIPL: ganglion cell and inner plexiform layer, INL – inner nuclear layer, mRNFL – macular retinal nerve fibre layer, N/T ratio – nasal to temporal ratio, OCT: Optical coherence tomography, ORL: outer retinal layers from outer plexiform layer to Bruch’s membrane, PMB: papillo-macular bundle, pRNFL: peripapillary retinal nerve fibre layer, RNFL-I – inferior retinal nerve fibre layer, RNFL-N – nasal retinal nerve fibre layer, RNFL-S – superior retinal nerve fibre layer, RNFL-T – temporal retinal nerve fibre layer, TMV: total macular volume (in mm³). (DOCX 20 kb) [file 12311_2020_1130_MOESM3_ESM.docx]

|  |  | Ring scan parameters | | | | | | Layer Segmentation Analysis | | | | |
| --- | --- | --- | --- | --- | --- | --- | --- | --- | --- | --- | --- | --- |
|  | Eye | pRNFL | PMB | RNFL-  I | RNFL-S | RNFL-N | RNFL-T | TMV [mm³] | mRNFL | GCIPL | INL | ORL |
| Patient 1 | OD | 103 | 55 | 144 | 122 | 78 | 69 | 9.38 | 38.9 | 84.2 | 42.4 | 166.2 |
|  | OS | 104 | 50 | 125 | 141 | 87 | 64 | 9.38 | 38.2 | 84.5 | 42.1 | 166.9 |
| Patient 2 | OD | 99 | 41 | 139 | 117 | 90 | 52 | 8.85 | 30.1 | 71.4 | 34.3 | 177.2 |
|  | OS | 97 | 41 | 128 | 117 | 91 | 52 | 8.86 | 31.1 | 72.5 | 34.0 | 175.8 |
| Patient 3 | OD | 100 | 65 | 132 | 125 | 63 | 79 | 9.01 | 36.1 | 72.2 | 34.0 | 176.5 |
|  | OS | 101 | 51 | 116 | 151 | 69 | 66 | 9.02 | 34.7 | 72.9 | 34.7 | 176.8 |
| Patient 4 | OD | 86 | 50 | 119 | 92 | 68 | 64 | 8.00 | 31.5 | 63.3 | 30.1 | 158.1 |
|  | OS | 90 | 53 | 115 | 105 | 79 | 62 | 8.26 | 34.3 | 65.8 | 31.5 | 160.6 |
| Patient 5 | OD | 106 | 62 | 145 | 121 | 74 | 85 | 8.92 | 36.1 | 77.5 | 34.3 | 167.6 |
|  | OS | 112 | 47 | 153 | 133 | 96 | 67 | 8.98 | 36.4 | 78.2 | 34.7 | 168.4 |
| Patient 6 | OD | 95 | 49 | 134 | 112 | 76 | 58 | 8.61 | 31.5 | 61.9 | 33.2 | 177.9 |
|  | OS | 99 | 48 | 136 | 119 | 84 | 56 | 8.66 | 31.8 | 63.0 | 33.6 | 177.9 |
| Patient 7 | OD | 110 | 44 | 164 | 114 | 104 | 59 | 8.89 | 32.5 | 75.3 | 33.2 | 173.3 |
|  | OS | 106 | 44 | 139 | 138 | 90 | 60 | 9.06 | 34.7 | 77.5 | 34.3 | 174 |
| Patient 8 | OD | 114 | 60 | 178 | 128 | 71 | 79 | 8.73 | 32.2 | 75.3 | 34.0 | 167.3 |
|  | OS | 109 | 50 | 160 | 136 | 76 | 66 | 8.66 | 31.5 | 74.6 | 35.0 | 165.2 |
| Patient 9 | OD | 77 | 45 | 110 | 79 | 66 | 56 | 8.40 | 30.1 | 64.7 | 34.7 | 167.6 |
|  | OS | 79 | 37 | 99 | 93 | 71 | 50 | 8.55 | 31.1 | 69.3 | 34.7 | 167.3 |
| Patient 10 | OD | 109 | 82 | 127 | 129 | 69 | 112 | 8.90 | 38.2 | 77.1 | 35.7 | 163.8 |
|  | OS | 110 | 63 | 130 | 142 | 80 | 87 | 8.89 | 38.9 | 76.7 | 35.4 | 163.4 |
| Patient 11 | OD | 103 | 59 | 136 | 114 | 87 | 77 | 8.59 | 39.3 | 66.5 | 31.5 | 166.6 |
|  | OS | 103 | 45 | 143 | 120 | 91 | 60 | 8.46 | 36.4 | 65.8 | 31.1 | 165.9 |
| Patient 12 | OD | 107 | 64 | 163 | 112 | 73 | 79 | 8.93 | 35.4 | 71.8 | 35.0 | 173.7 |
|  | OS | 107 | 52 | 145 | 138 | 80 | 67 | 8.99 | 36.1 | 72.5 | 34.7 | 174.7 |
| Patient 13 | OD | 87 | 54 | 125 | 98 | 54 | 71 | 8.68 | 35.7 | 66.8 | 32.5 | 171.9 |
|  | OS | 87 | 51 | 128 | 94 | 60 | 67 | 8.65 | 34.0 | 67.2 | 32.9 | 171.9 |
| Patient 14 | OD | 97 | 56 | 133 | 109 | 82 | 64 | 8.69 | 36.4 | 67.9 | 32.9 | 170.1 |
|  | OS | 108 | 49 | 143 | 131 | 98 | 61 | 8.92 | 37.5 | 70.4 | 32.5 | 175.1 |
