# Supplementary material for: Investigation of Visual System Involvement in Spinocerebellar Ataxia Type 14
Source: Cerebellum. 2020 Apr 27;19(4):469–82. doi: 10.1007/s12311-020-01130-w (PMC7351844; doi:10.1007/s12311-020-01130-w)
Supplement: Supplementary file 3 [file 12311_2020_1130_MOESM2_ESM.docx]

Patient	Age	Family	Genetic variant	Eye	Fundus	logMAR monocular	logMAR binocular	FACT binocular	Self-reported ophthalmologic history	SARA Score	Disease duration	Statistically evaluated
P1	51	1	G23E	OD		-0.04	-0.04	1.88		10.5	22	Yes
				OS		-0.12						
P2	40	1	G23E	OD	calcifying exsudates	-0.16	-0.06	1.99		7.5	15	Yes
				OS		0.14						
P3	31	2	C66Y	OD	remarkable tortuosity	-0.10	-0.18	2.17		9.5	18	Yes
				OS	remarkable tortuosity	-0.10			status post eye infection			
P4	60	2	C66Y	OD		0.24	0.12	1.94	ocular pain	20.5	12	Yes
				OS		0.14			ocular pain			
P5	63	2	C66Y	OD		-0.10	-0.18	1.97		9.5	13	Yes
				OS		-0.10						
P6	51	singular	C66Y	OD	drusen	0.04	-0.08	2		13.5	5	Yes
				OS		0.16						
P7	68	3	82-84delTFQ	OD		-0.10	-0.1	2.03		14.5	32	Yes
				OS		-0.02						
P8	60	3	82-84delTFQ	OD		-0.20	0.22	1.7		11	17	Yes
				OS		0.24						
P9	62	singular	F113-C114delinsC	OD		0.02	-0.08	2.02	glaucoma/double vision	9.5	9	No
				OS		0.02			glaucoma/double vision			(glaucoma)
P10	43	singular	H116P	OD		0.16	-0.04	1.63		11	42	Yes
				OS		0.06						
P11	70	singular	G123A	OD		0.12	0.14	1.79		11	33	Yes
				OS		0.30						
P12	55	4	C131R	OD		0.00	-0.08	2.02	double vision	11.5	44	Yes
				OS		0.02						
P13	29	4	C131R	OD		0.12	-0.04	1.9		6.5	0	Yes
				OS		0.14						
P14	50	singular	R634H	OD	pigment epitheliopathy	-0.06	-0.18	2.25		14	5	No
				OS	pigment epitheliopathy	-0.16						(VUS)
